# Interactions of neuroimmune signaling and glutamate plasticity in addiction

**DOI:** 10.1186/s12974-021-02072-8

**Published:** 2021-02-21

**Authors:** Cassandra D. Gipson, Scott Rawls, Michael D. Scofield, Benjamin M. Siemsen, Emma O. Bondy, Erin E. Maher

**Affiliations:** 1 Department of Family and Community Medicine, University of Kentucky, 741 S. Limestone, BBSRB, Room 363, Lexington, KY 40536-0509 USA; 2 Department of Pharmacology, Lewis Katz School of Medicine, Temple University, Philadelphia, USA; 3 Department of Anesthesiology, Medical University of South Carolina, Charleston, USA; 4 Department of Neuroscience, Medical University of South Carolina, Charleston, USA

**Keywords:** Neuroimmune, Glutamate, Microglia, Astroglia, Addiction

## Abstract

Chronic use of drugs of abuse affects neuroimmune signaling; however, there are still many open questions regarding the interactions between neuroimmune mechanisms and substance use disorders (SUDs). Further, chronic use of drugs of abuse can induce glutamatergic changes in the brain, but the relationship between the glutamate system and neuroimmune signaling in addiction is not well understood. Therefore, the purpose of this review is to bring into focus the role of neuroimmune signaling and its interactions with the glutamate system following chronic drug use, and how this may guide pharmacotherapeutic treatment strategies for SUDs. In this review, we first describe neuroimmune mechanisms that may be linked to aberrant glutamate signaling in addiction. We focus specifically on the nuclear factor-kappa B (NF-κB) pathway, a potentially important neuroimmune mechanism that may be a key player in driving drug-seeking behavior. We highlight the importance of astroglial-microglial crosstalk, and how this interacts with known glutamatergic dysregulations in addiction. Then, we describe the importance of studying non-neuronal cells with unprecedented precision because understanding structure-function relationships in these cells is critical in understanding their role in addiction neurobiology. Here we propose a working model of neuroimmune-glutamate interactions that underlie drug use motivation, which we argue may aid strategies for small molecule drug development to treat substance use disorders. Together, the synthesis of this review shows that interactions between glutamate and neuroimmune signaling may play an important and understudied role in addiction processes and may be critical in developing more efficacious pharmacotherapies to treat SUDs.

## Introduction

Mechanisms of neuroimmune signaling have been linked to stress [49, 171, 318], as well as neurodegenerative (e.g., Alzheimer’s disease [52, 215]) and neuropsychiatric disorders (e.g., depression [133]; nicotine and alcohol use disorder [46, 70, 228, 246, 258, 259]). Studies have shown that inflammation can significantly alter motivated behavior in the short term which can be adaptive (e.g., sickness [74]), but can also be maladaptive, such as in major depressive disorder where reductions in activity [331] and responses to rewards [85] are associated with elevated immune signals such as tumor necrosis factor alpha (TNFα). Much less is known in the substance use disorder (SUD) field regarding peripheral or central immune contributions to maladaptive drug use, though recent studies show that drugs of abuse interact with neuroimmune processes. These interactions may drive the pathological motivation to seek drugs, and thus, neuroimmunomodulation of drug-motivated behavior is a novel and exciting frontier with the potential to reshape our current understanding of the neurobiological mechanisms underlying drug addiction vulnerability.

Given that the role of neuroimmune signaling in addiction is a relatively new area of research, little is known regarding specific mechanisms that drive drug use. Within the field of addiction, it has long been established that dysregulated drug use is associated with a transition from goal-directed to habitual drug-seeking motivation [88, 194], and this is a cardinal characteristic of SUD. Further, although drugs of abuse have different direct mechanisms of action, there are conserved neurobiological changes across drug classes that likely interact with neuroimmune processes following both chronic and subchronic exposure. The purpose of this review is to bring into focus the role of neuroimmune signaling in driving drug addiction motivation, and based on what we *do* know regarding interactions of neuroimmune mechanisms and chronic drug use, we outline potentially critical interactions with known drug-induced changes in the glutamate system. Here, we will begin by outlining potentially important neuroimmune processes with regard to SUD and how they (1) interact with glutamate signaling and (2) influence motivated drug-seeking behavior. Notably, the immune system is comprised of both central and peripheral immune systems, thus we will briefly describe the contributions of these to physiology, and how that may contribute to neuroimmune functions. Importantly, we will describe the well-characterized role of glutamate homeostasis in drug addiction and bring into focus how neuroimmune processes may interact with this system to influence drug-motivated behavior. Given the importance of tailoring drug discovery efforts with sex specificity as well as the recent mandate by the National Institutes of Health to include both sexes [303], we will also describe sexual dimorphisms in neuroimmune signaling and their potential effects on the corticolimbic reward circuit. The goal of this section is to highlight the dearth of knowledge regarding how ovarian hormones may interact with neuroimmune signaling and glutamate homeostasis in substance use disorders (SUDs) in a sex-specific fashion (as noted in another recent review; see [108]). Next, we will focus on microglia-astroglia communication and detail the current state of the field with regard to available techniques to study neuroimmune signaling. This section highlights new technology that allows for more intricate connections to be made between structure and function of astroglia and microglia. Finally, to bring this review into context with current pharmacotherapies under examination to treat SUDs, we will describe small molecule drug therapies that restore glutamate homeostasis and may also exert anti-inflammatory effects to curb drug-seeking behavior. Taken together, the goal of this review is to demonstrate how neuroimmune and glutamatergic signaling may interact within the reward pathway to drive drug use vulnerability.

## Striking a balance: neuroimmune signaling

The innate immune network within the central nervous system (CNS) is critical to reducing neuronal damage in response to environmental neuroimmune insults, but the immune network itself can also lead to destructive effects. Glial cells, including microglia and astroglia, as well as other cell types, provide support and nutrients to neurons. Further, these cells protect the CNS from injury by upregulation of neuroimmune processes [18]. However, glial overactivation can lead to severe neuronal damage which can further exacerbate neuroinflammation and neurodegeneration. It is not just glial overactivation that can damage the nervous system; many neurotransmitters and signaling molecules are toxic at high levels (e.g., excitotoxicity induced by excessive glutamate levels [179]). This toxicity itself has been proposed as a potential mechanism for reducing colonization by microbes that might otherwise hijack the brain [76]. But, as with many defensive measures, excessive levels of molecules such as neurotransmitters come with costs—in this case to the host, as these excessive levels can compromise normal brain and immune function. There must be a balance between neuroprotection and neurotoxicity [68, 134].

Neuroinflammation within the CNS occurs in response to injury by immunocompetent cells which also communicate with the peripheral immune system. For example, immune cells from the periphery can infiltrate the CNS in response to injury [167] or stress. One preclinical example of this is a study which found that bone marrow-derived microglia infiltrate the hippocampus of mice following chronic foot-shock stress [45]. Interestingly, in this study, these monocytes developed microglia-like characteristics, including ramification morphology and were Iba-1-positive but glial fibrillary acidic protein (GFAP)-negative. Another study found infiltration of bone marrow-derived monocytes in the paraventricular nucleus of the hypothalamus following a stress paradigm in mice [11], demonstrating peripheral cell recruitment is an important mechanism in response to a stressor [210].

Neuroimmune signals including cytokines and neurotrophic factors are also not exclusive to the central or peripheral immune systems as there is a large amount of overlap between the two [134]. Neurotrophic factors are a family of proteins which play a critical role in normal CNS development within vertebrate animals. These factors regulate neuronal survival as well as growth of dendritic arbors and plasticity [25, 193]. Further, neurotrophic factors, such as brain-derived neurotrophic factor (BDNF) and glial cell line-derived neurotrophic factor (GDNF), play important roles in synaptic plasticity and immune cell functions including migration, activation, and differentiation [134, 311].

Cytokines can serve protective or destructive roles during neuroinflammation. For example, interleukin-37 (IL-37) has been found to inhibit innate inflammation both in vitro and in vivo. Pharmacotherapeutic targeting of IL-37 has been conducted for the treatment of multiple inflammatory diseases such as asthma, fibromyalgia, and chronic inflammatory disease [15, 200, 211], but has not yet been studied in the context of SUDs. Although not known, it is hypothesized the mechanisms by which IL-37 exerts its anti-inflammatory actions may be through inhibition of the mammalian target of rapamycin (mTOR [186]), or through inhibition of inflammasome activation [221]. The mTOR pathway has been implicated in addiction processes, specifically with psychostimulants [17, 320]. Conversely, pro-inflammatory cytokines are pleiotropic, whereby a given cytokine can trigger proliferation in one cell type but then can lead to inhibition of growth in another cell type. Pro-inflammatory cytokines are produced in response to tissue injury or neurodegeneration within the CNS by tissue-invading leukocytes. As well, glial cells contain inflammasomes which are cytosolic multiprotein complexes that activate pro-inflammatory caspases (mainly caspase 1 [253]). Within the addiction field, methamphetamine exposure has been found to activate inflammasomes [335, 343]. Activation of these caspases leads to release of pro-inflammatory cytokines and an inflammatory response. It should be noted that the contribution of some mediators to neuroinflammation can vary depending on when they are administered during the course of disease. Within the addiction field, there is evidence for this phenomenon as fractalkine (also termed CX3CL1) can serve as a neuronal off-signal to maintain the anti-inflammatory state of microglia; however, there is also evidence of it serving a pro-inflammatory role as it has been to mediate nicotine withdrawal-induced hyperalgesia in rats [80].

## Central and peripheral immune interactions: potential contributions to addiction neurobiology

As noted above, the immune system is comprised of both central and peripheral mechanisms, which have until more recently been studied in isolation. As such, immunology has only recently recognized that the CNS is not immunoprivileged, but rather has interactions with the immune system [73]. Here we briefly discuss interactions with and distinctions between central and peripheral immune mechanisms.

One way in which central and peripheral immune mechanisms interact is through neurotransmitters, which interact with peripheral immune cells and regulate their function. For example, human T cells, which are produced in bone marrow and are critical for eradication of infections and cancer, express both ionotropic and metabotropic glutamate receptors. As such, glutamate plays critical roles in tissue other than brain, including heart, kidney, intestine, lungs, and ovaries, among others [104, 131, 230]. In the next section, we will delve into the critical role of glutamate in the brain reward pathway to motivated drug seeking.

Other neurotransmitters typically studied within the CNS also appear to interact with peripheral immune systems. For example, γ-aminobutyric acid (GABA), the main neuroinhibitory transmitter in the brain, is also present in peripheral tissues such as pituitary, ovaries, placenta, among others [111]. Importantly, GABA mRNA has been identified in human peripheral blood mononuclear cells [3] and GABA itself has been detected in peripheral blood monocyte-derived macrophages [296]. Functionally, GABA can activate or reduce secretion of cytokines [312]. In addition to GABA, serotonin has been shown to interact with peripheral immune cells and plays a critical role in immune cell recruitment [183], macrophages that express the serotonin transporter (SERT [144]). Because neurotransmitter systems typically studied within the CNS have long been shown to play critical roles in peripheral immune functions, and these same neurotransmitter systems have been heavily implicated in addiction neurobiology [151, 172], there is biological plausibility for reciprocal relationships of central and peripheral immune mechanisms in addiction.

Given the focus of this review on microglia as critical mediators of motivated drug seeking in addiction through interactions with the astroglia-neuron glutamate synapse, it is important to highlight that these cells are distinct from peripheral macrophages. Both microglia and peripheral macrophages come from primitive macrophages; however, microglia come from yolk sac progenitors and migrate to the developing CNS before the closure of the blood brain barrier (BBB; [106]). Microglia do share some genes with other mononuclear microphages; however, there are some transcripts that are highly enriched in microglia such as CX3CR1, P2RY12, SOCS3, GPR34, TMEM119, and SALL1 among others (some of which are considered exclusively markers of microglia; see [185]). Importantly, only those cells from yolk sac origin fully attain microglial identity, making them distinct from peripheral macrophages which can express some microglial genes when settled in the brain [28].

## Toll-like receptors and their role in addiction

Emerging evidence indicates a close link between addiction and dysregulation of central immune pathways, which includes activation of microglia through specific receptors. Immune signaling involves a number of receptors within a large class of receptors termed “pattern recognition receptors” (PRRs), which are categorized by the ligands which bind to them, their cellular localization, and the outcomes, or consequences, following their activation [145]. One class of pattern recognition receptors (PPRs) that has been implicated in the neuropathology of addiction is toll-like receptors (TLRs [87, 141, 235, 327, 341, 347];), and the first PPRs identified and have since been established as critical mediators of innate immune signaling [162]. TLRs are expressed on microglia, and upon activation, these receptors trigger intracellular signaling cascades that induce immune responses [181]. Importantly, stimulation of TLRs activates signaling pathways resulting in elevated inflammatory cytokines, including interleukin-1, interleukin-6, and interleukin-8 (IL-1, IL-6, and IL-8) [206]; these cytokines are transcribed through TLR4-mediated activation of the nuclear factor-kappa B (NF-κB) pathway. This is important because pro-inflammatory neuroimmune signaling within the brain regulates AMPA and GABA receptor trafficking [293], which may indicate important links between pro-inflammatory signaling induced by TLR activation, glutamate plasticity, and addiction.

As mentioned above, TLRs are highly expressed on microglia [128, 244]. Further, TLRs have been heavily implicated in addiction processes across drug classes. For example, the alcohol field has identified neuroimmune signaling, and specifically TLRs, as a critical component of alcohol use disorder [64–66]. One study showed that binge ethanol exposure during adolescence promotes alterations in synaptic plasticity, which was associated with alcohol preference, an effect that was not observed in TLR4 knockout mice [219]. Another study found that TLR4 activation may contribute to disruption of BBB integrity following ethanol exposure via the drinking in the dark paradigm [256]. Additionally, there is evidence for the role for TLR4 in polysubstance use, as alcohol and nicotine use vulnerability was shown to be modulated by TLR4 in the ventral tegmental area [22]. Other drugs of abuse, such as cocaine and opioids, have been associated with TLR activation within the nucleus accumbens (NA [140, 347]), another key node of the reward pathway. One study showed that TLR3 inhibition as well as deficiency reduces cocaine conditioned place preference (CPP), locomotor activity, and cocaine self-administration in mice. Further, TLR3 inhibition reversed cocaine-induced upregulation of key proteins involved in activation of the NF-κB pathway, such as phospho-NF-κB, p65, IκB kinase (IKK), and p-IκBα [347].

As mentioned above, TLRs interact critically with the NF-κB pathway in models of addiction. Studies have shown involvement of TLR4 in addiction-related behaviors. Specifically, one study found that deficiency of TLR4 via a knockout mouse model was associated with decreases in long-term depression (mediated through *N*-methyl-d-aspartic acid (NMDA) receptors within the NAcore) as well as attenuated cocaine conditioned place preference (CPP [159];). This same study also showed that these receptors are expressed primarily on microglia within the NAcore. This study is critical as it links TLRs with glutamatergic plasticity mechanisms within the NAcore, which have been shown to be critical neurobiological alterations induced by chronic use of drugs of abuse [269]. Another study found that cocaine CPP and self-administration is disrupted by blocking cocaine-induced changes in TLR4 activation [235]. This study also showed that NAcore dopamine release is suppressed through a TLR4-mediated mechanism. Together, these results demonstrate that TLR signaling, and its activation of the NF-κB pathway, is critical in SUDs across various classes of drugs of abuse. Below we will delve further into connections between glutamatergic signaling, TLRs and the NF-κB pathway, and addiction.

## Developing an understanding of the interactions between neuroimmune signaling and glutamate in addiction

Decades of studies have provided a clear link between altered plasticity at glutamatergic synapses in the NAcore and relapse of drug seeking following exposure to several drugs of abuse, including cocaine, heroin, methamphetamine, nicotine, and ethanol [109, 110, 117, 151, 177, 224, 277]. Astroglia are critical regulators of excitatory transmission in the NAcore and do so via the homeostatic regulation of extracellular glutamate levels. Astroglia maintain basal levels of glutamate in the NAcore via glutamate release through the cystine-glutamate antiporter (xCT), a glial system whereby extracellular cysteine is exchanged for intracellular glutamate [21]. This release provides a significant proportion of the overall basal glutamate tone on presynaptic metabotropic glutamate receptors (e.g., mGluR_2/3_), limiting evoked glutamate release. Decreased function of xCT ([20] a), and thus decreased levels of basal glutamate [29], in the NAcore is a cornerstone of addiction biology and is directly linked to dysfunctional glutamate homeostasis underlying relapse vulnerability [151]. Astroglia clear synaptic glutamate via activity of the glutamate transporter (GLT-1 [301]). The largely conserved drug-induced decrease in GLT-1 results in an inability to clear evoked glutamate release arising from cortical terminals when animals are undergoing cue- or drug prime-induced seeking for drugs such as cocaine [205, 283] and heroin [177, 272]. Thus, astrocytic dysfunction in regulating basal glutamate, as well as clearance of synaptic glutamate, has been established as a primary mechanism underlying addiction pathology and cue-induced drug-seeking behavior [270].

**Fig. 1:**
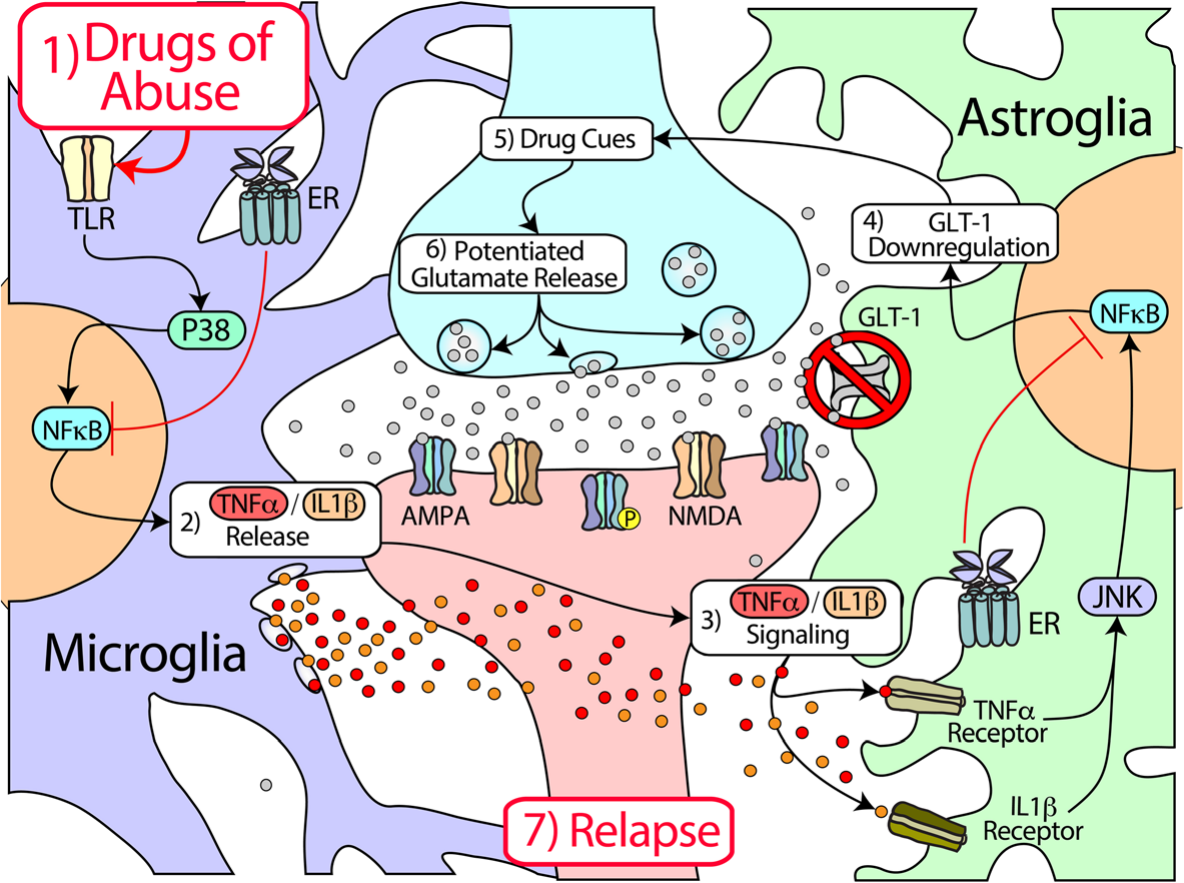
Hypothesized nucleus accumbens neuroimmune-glutamate interactions in addiction. Drugs of abuse (1) activate TLRs, which (2) triggers the NF-κB signaling pathway within microglial cells through activation of P38, which is expressed in activated microglia. Microglia then release pro-inflammatory cytokines such as TNFα and IL-1β. These cytokines then (3) bind to their receptors (TNFR, IL-1βR) on astroglia, which activates NF-κB through JNK pathways. Specifically, binding of these cytokines leads to activation of the IKK, c-Jun N-terminal kinase (JNK), and p38 MAPK, which leads to activation of the transcription factor NF-κB. This then leads to (4) repression of GLT-1 transcription and ultimately downregulation of the GLT-1 transporter, as TNFα negatively regulates EAAT2 transcription. Downregulation of GLT-1 protein results in an inability of astroglia to clear excess glutamate from the synapse during reinstated drug seeking (5). Following exposure to drug-associated cues, (6) glutamate release from cortical afferents into the nucleus accumbens is potentiated, leading to (7) activation of ionotropic glutamate receptors (e.g., AMPA, NMDA), rapid, transient post-synaptic plasticity, and relapse. In females, estrogen receptors (ERs) are located on various cell types including microglia and astroglia, and can directly inhibit NF-κB. TLR = toll-like receptor; P38 = p38 mitogen-activated protein kinase (MAPK); NF-κB = nuclear factor-kappa B; TNFα = tumor necrosis factor alpha; IL = interleukin; GLT-1 = glutamate transporter-1

As stated, glutamate is released from prelimbic cortical afferents in the NAcore during drug-seeking behavior ([205, 283, 291]; also see increased glutamate in humans following 12 h of alcohol withdrawal, [48]). Glutamate binds post-synaptically to α-amino-3-hydroxy-5-methyl-4-isoxazolepropionic acid (AMPA) and NMDA receptors, and enters the extracellular space due to downregulation of GLT-1. The net result is changes in post-synaptic plasticity [110, 272]. The ability of cues to drive drug seeking, at least for the case of cocaine, heroin, and nicotine, has been attributed to a transient increase in synaptic potentiation at glutamatergic synapses in the NAcore [109]. Transient synaptic potentiation is characterized by increased AMPA/NMDA ratios and elevated dendritic spine head diameter in NAcore medium spiny neurons (MSNs), which requires activation of matrix metalloproteinases (MMPs) through S-nitrosylation [281], and thus activation of NAcore nitrergic interneurons [283] to release nitric oxide (NO [276]). From the above findings, it can be concluded that glutamate overflow onto NAcore nitrergic interneurons, due to dysfunctional astroglia clearance, is likely a primary mechanism whereby cues associated with drugs of abuse can drive relapse through altered plasticity at NAcore glutamatergic synapses on MSNs. Although the majority of the NAcore signaling cascades leading to the induction of cued seeking have been well characterized, it is unclear how GLT-1 and xCT are dysregulated by drug exposure. We posit that disturbances in neuroimmune signaling, specifically drug-induced alterations in microglia and their communication with astroglia and neurons, are important mediators of such adaptations (see Fig. 1). Importantly, microglia-neuron-astroglia interactions as we propose in this figure have not been well characterized as a function of withdrawal timepoint, nor have they been well defined across different drugs of abuse. These are important future research directions.

As mentioned in the section above, several drugs of abuse, but particularly opioids, have been shown to directly activate microglia through TLR4 and/or mu (μ) opioid receptors on resident microglia [199, 325], whereby μ opioid receptor activation accentuates lipopolysaccharide (LPS)-induced microglia activation-mediated NF-kB signaling [103, 141]. Importantly, LPS is both a microbial product and can be exogenously infused to induce sepsis, and subsequently, an immune response. In addition to neurons and astroglia, microglia also express a broad range of glutamate receptors, as well as glutamate transporters [90, 227]. GLT-1 has been shown to be positively regulated by the uptake of neuronally derived exosomes enriched in microRNA (miR)-124, and a recent study found that the number of exosomes is reduced in both astroglia and microglia following cocaine self-administration [209], suggesting that cocaine experience alters the interplay between neurons and glial cells which may be important for subsequent relapse vulnerability. Importantly, miR-124 functions in microglia to promote anti-inflammatory cytokine release, and overexpression of miR-124 in the striatum suppresses cocaine-mediated microglia activation and hyperactivity by preventing cocaine’s ability to upregulate TLR4 [248], further linking microglia activity to altered behavioral patterns evoked by drug exposure. As described above, one consequence of TLR4 activation is elevated pro-inflammatory cytokine release, particularly TNFα, which can have a profound influence on both neurons and astroglia. Because reduced GLT-1 expression might depend on increased transcription of inflammatory factors due to upregulation of miRNAs, these signaling pathways must be considered in neural-glial communication and may impact glutamate homeostasis.

Microglia shape neuronal communication through cytokine release and neuroimmune signaling, with disruptions of these processes described in several pathological conditions [31, 89, 163]. Using a genetically modified Mg^PTX^ mouse model to inhibit Gi in microglia, one new study found that Gi-dependent microglia dynamics may block neuronal hyperexcitability, and this may be important in disease [212]. In accordance with the well-established role for bidirectional communication between microglia and neurons in the regulation of neuronal structural and synaptic plasticity [36, 328, 333], microglia-mediated TNFα release increases NR1 surface expression and NMDA-mediated Ca^2+^ influx and evokes excitatory post-synaptic currents in hippocampal neurons [330]. Alternatively, TNFα has been shown to lead to a rapid insertion of Ca^2+^-permeable AMPA receptors in hippocampal pyramidal neurons [293]. This latter point is particularly relevant because long access cocaine self-administration followed by withdrawal increases Ca^2 + -^permeable AMPA receptor accumulation in NAcore MSNs which is directly linked to craving following extended withdrawal [61, 203]. Though a rise in TNFα levels and a decrease in AMPA/NMDA ratio is observed after chronic methamphetamine administration [216], it was found TNFα-knockout mice administered more methamphetamine than controls [190, 216, 336]. Moreover, cocaine-induced increases in TNFα in the NAcore have been shown to be *protective* against cocaine locomotor sensitization and do so by *decreasing* AMPA/NMDA ratios in D1 MSNs [184]. It is likely that differences in experimental design, including contingent versus non-contingent cocaine administration, differentially alter the ability of cocaine to engage TNFα. Moreover, the degree to which different drugs of abuse engage microglia activation via TLR4 is likely an important factor in the differential pathology underlying psychostimulants relative to opiate-based drugs of abuse.

In the healthy brain, TNFα release from microglia is critical for regulating synaptic transmission in several brain regions. However, this basal amount exists in the 100–200 pM range [261]. At these low concentrations, TNFα is thought to be permissive for effective glio-transmission (i.e., release of glutamate from astroglia). However, at high concentrations (~ 100 nM), TNFα leads to massive glutamate release from astroglia [238]. To put this in perspective, when TNFα concentrations reach a plateau, TNFα is able to increase astroglia glutamate release threefold higher than any recorded GPCR agonist [34]. Thus, TNFα can act as a potent signaling molecule linking microglia to astroglia, and ultimately neurons. Accordingly, basal microglial release of TNFα acts as a prerequisite for circuits to undergo synaptic scaling, a phenomenon that can be described as an increase or decrease in single-cell synaptic strength or excitability in response to circuit-wide neuronal activity [294]. Cultured NAcore MSNs undergo synaptic scaling in their natural state, and the ability to do so is altered by prolonged addition of dopamine [299]. Microglial regulation of synaptic scaling is thought to be engaged by altering astroglia-mediated activation of metabotropic glutamate receptors [242], a cellular mechanism linked to relapse vulnerability across several drugs of abuse including cocaine, nicotine, and opioids, among others [269, 270]. Thus, it is likely that a switch in the ability of TNFα to either allow for normal synaptic function or contribute to aberrant plasticity, or altered synaptic scaling, is likely due to changes in the local concentration of TNFα as a result of neuroimmune insult or chronic activation of microglia.

Apart from the well-described ability of TLR4 activation, and the subsequent release of TNFα, to engage altered synaptic function, there is also a role for microglia in releasing NO [63] and activating matrix metalloproteinase 9 (MMP9 [67]) through alternative pathways, both of which occur upstream of synaptic glutamate overflow in cue-induced cocaine seeking [281]. While the source of MM9 is currently under debate [282], some studies have shown that methamphetamine is associated with release of MMP9 and MMP2 through endothelial cells (e.g., see [91, 92]). One other potential source is resident microglia. In cultured microglia, activation of TLR2 increases microglial MMP9, which is NO-dependent [16]. Although neuronal nitric oxide synthase (nNOS) activation, caused by mGluR5 activation, results in NO production necessary for cue-induced cocaine seeking, fluctuations in microglial NO release could contribute to the neuronal pool [276, 283]. This would lead to additive effects in MMP9 S-nitrosylation, consequently altering MSN structural and functional synaptic plasticity. Given that daily cocaine injections increase TLR2 phosphorylation and TNFα in the striatum [187], it is likely that the increase in MMP9 could be due to activation of NAcore microglia through the TLR2 pathway during cue-induced seeking, although this has yet to be shown experimentally.

There is a wealth of data indicating that brain regions other than the striatum, particularly the prelimbic component of the medial prefrontal cortex (mPFC), undergo morphological and electrophysiological adaptations after cocaine exposure. For example, 1 week of abstinence from cocaine self-administration decreases the density of dendritic spines in prelimbic neurons projecting to the NAcore [275]. Moreover, compulsive drug seeking following extended cocaine self-administration, characterized by persistent cocaine seeking despite foot shocks, decreases the intrinsic excitability of prelimbic output neurons [56], which is paralleled by clinical studies revealing decreased glucose metabolism in frontal cortical regions of human cocaine abusers [113]. Induction of neuroimmune signaling by acute LPS injections leads to rapid microglia activation, but a delayed decrease in the density of dendritic spines in vivo [169]. Using a mouse model of myocardial infarction, increased TNFα signaling has been linked to a loss of cortical dendritic spines [208]. In the field of addiction, alongside the pervasive increase in the expression of inflammatory signaling factors resulting from activation of the NF-kB pathway seen across multiple drugs of abuse, dendritic spine loss has also been observed following chronic ethanol, cocaine and morphine exposure [114, 214, 290]. Microglia associate with synapses, and dendritic spines, in an experience-dependent manner whereby they can engulf presynaptic connections at spines [328]. This is particularly well-established as a fundamental mechanism of circuit remodeling during development, but has also been shown to persist into adulthood at select synapses [115]. We posit that this learning-dependent modification of neuronal networks may be one mechanism whereby the loss of dendritic spines, and subsequent hypofrontality observed following cocaine exposure, may be due to aberrant activation of microglia in the adult cortex and thus an inability to effectively regulate striatal circuitry associated with motor activation in response to drug-associated stimuli [152].

## An emerging mechanism: NF-κB signaling and addiction

As described in detail above, disruptions in glutamate signaling have been well characterized following chronic use of and relapse to drugs of abuse (for an in-depth review of the topic, see [269]). Although glutamatergic neuroadaptations have long been a focus of the addiction field, the molecular mechanisms by which drugs of abuse alter glutamatergic signaling remain incompletely understood. There are known interactions between TNFα and the NF-κB pathway with glutamate synaptic communication, and the NF-κB pathway has been extensively studied in the areas of stress and addiction. Activation of this pathway appears to interact with glutamate signaling in addiction models [228, 278, 280, 297], and as such, the NF-κB pathway may prove critical in driving aberrant glutamate changes in addiction. Here we present a case for the importance of the NF-κB pathway and its activation by TNFα in driving dysregulated glutamate signaling in addiction. Following discussion of NF-κB-glutamate interactions and how this may be critical in driving addiction-related behavior, we next highlight that there may be sex-specific differences in how the NF-κB pathway is activated and involved in glutamate homeostasis in addiction within the next section.

Neuroimmune signaling has been shown to interact with neurotransmission and, specifically, has an important role in maintaining normal glutamatergic signaling in order to prevent excitotoxicity [34, 122, 300]. Importantly, GLT-1 transports > 90% of glutamate out of the synapse [126] and is downregulated by several drugs of abuse including cocaine, alcohol, nicotine, and heroin [110, 254, 255, 272]. Please note that when discussing the protein below, the term used will be GLT-1; when discussing the gene, the term used will be Excitatory Amino Acid Transporter-2 or EAAT2. Next we will describe how TNFα controls EAAT2 gene transcription and subsequent expression of GLT-1 protein, and how this is relevant for dysregulation of glutamate homeostasis in addiction.

Impaired glutamate uptake by glia can lead to cell death due to overactivation of glutamate receptors [59]; this mechanism has been linked to Alzheimer’s disease and amyotrophic lateral sclerosis among others [81]. The EAAT2 gene is induced by epidermal growth factor and is downregulated by tumor necrosis factor α (TNFα) through the NF-κB pathway. Specifically, p65 binding to the -583 site of the EAAT2 promoter is increased following activation by TNFα. Further, EAAT2 expression required NF-κB as determined by mutation of the -583 site in which mutation impaired constitutive activation of EAAT2 [278]. Taken together, the results from this previous work and Sitcheran’s comprehensive study indicate that NF-κB is essential for basal activation of EAAT2, and TNFα negatively regulates EAAT2. This is important because it shows that TNFα and the NF-κB pathway interact with known glutamate dysregulations that are disrupted by addiction and relapse (e.g., see [110, 254, 255, 272]).

As mentioned above, neuroinflammation due to drugs of abuse appears to be mediated in large part by the NF-κB pathway [228, 259] and is highly conserved across various species and cell types [105]. NF-κB signaling is heavily involved in learning and memory processes [4, 153] and plays a role in dendritic spine morphological changes in response to cocaine [7]. As mentioned above, TNFα activates NF-κB signaling, and the NF-κB pathway is involved in learning, memory, and synaptic plasticity [4, 153, 207, 237, 293, 294]. Accordingly, TNFα signaling and the NF-κB pathway may underlie drug-induced alterations in synaptic plasticity and drug-seeking behavior. A recent study from our lab found an increase in soluble TNFα expression within the nucleus accumbens core (NAcore) during both nicotine withdrawal and after 15 min of cue-induced nicotine-seeking behavior. Further, inhibition of NF-κB via viral gene transfer of a dominant-negative form of IKK inhibited nicotine-seeking behavior and reduced GLT-1 expression, illustrating a critical role of NF-κB in driving nicotine-seeking behavior [228]. In the canonical signaling pathway, NF-κB heterodimers are maintained in an inhibited state within the cytoplasm through interactions with IκB (e.g., IκBα). Through ligand binding of extracellular signaling molecules (e.g., cytokines such as TNFα, IL-6, etc.) to their cell-surface receptors, adaptor proteins are recruited to the intracellular domain of the receptor, activating an IKK complex that phosphorylates IκB. This allows for the proteasomal degradation of IκB and translocation of NF-κB into the nucleus where it binds to DNA, alters transcription of its gene target [105], leading to secretion of the soluble homotrimer form of TNFα [57, 189, 324]. Finally, studies have demonstrated that the NF-κB pathway can lead to the activation of Rho GTPases, a class of proteins involved in synaptic plasticity and neuron morphological changes when exposed to psychoactive substances [78, 79].

NF-κB signaling occurs in multiple cell types including neurons, astroglia, and microglia [42, 148, 154, 156, 182, 289]. Within neurons, both pre- and post-synaptic components contain NF-κB signaling machinery [155]. NF-κB signaling occurs in astroglia [154], and astroglial activation drives microglial proliferation [241]. Microglial activation occurs in response to damage, and these cells are responsive to aberrant neurotransmission. Given that glutamate overflow occurs during drug-seeking behavior with various drugs of abuse including nicotine, cocaine, and heroin [110, 177, 283], there is biological feasibility for the role of microglia in the driving drug-seeking behavior. In further support, microglia express glutamate receptors, including AMPA [121, 234], NMDA [149], kainate, and group I [37], II [307], and III [306] metabotropic glutamate receptors (mGluRs; also see [119] for a review). Activation of these receptors by glutamate appears to contribute to microglial motility, activation state, and release of TNFα. Importantly, microglia also express GLT-1, and glutamate uptake by microglia occurs through this transporter [192, 227]. NF-κB signaling within microglia likely plays a critical role in microglial response to excessive glutamate release, which raises the possibility that microglia orient to the synapse during drug-seeking behavior in response to glutamate release within the NA. Further, this circuit may be critical in facilitating astroglia morphological alterations in response to drug seeking, as astroglia retract from the synapse during cocaine withdrawal [271], and return to the synapse during reinstatement of heroin seeking [175] following the same time course as a rapid increase in GLT-1 protein during cued nicotine seeking (within 15 min of reinstatement [228]).

There is evidence that drug self-administration leads to constitutive increases in pro-inflammatory (M1) and/or growth-promoting (M2) microglial expression; both are increased after alcohol [246]. Though the use of M1 and M2 classifications of microglial activation has been invalidated as demonstrated by these results (also see section “Interactions between microglia and astroglia” below for additional detail), the collective increase in phagocytic ability of microglia after alcohol exposure should be noted. Also exemplifying microglial-mediated inflammatory responses in SUDs is the binding by opioids and subsequent activation of myeloid differentiation factor 2 (MD-2)-TLR4 complex on microglia, resulting in increased expression of both anti- and pro-inflammatory signaling molecules via the NF-κB pathway [84]. It was further concluded that the degree of microglial activation was a significant predictor of morphine half-maximal antinociceptive dose (ED_50_) values, indicating a correlation between microglial inflammatory expression and opioid tolerance through NF-κB pathway [84]. Notably, females exhibit greater microglial activation compared to males following morphine exposure. Below, we will describe sexual dimorphisms in neuroimmune processes, which may contribute to sex differences in SUD vulnerability.

### Estrogen and the NF-κB pathway: a sex-specific role in addiction processes?

The prior section laid a foundation upon which further research should be conducted to fully characterize how the NF-κB pathway may be critically involved in addiction-related behavior. The goal of this section is to lay the groundwork for future studies aimed at understanding how steroidal hormones interact with the NF-κB pathway and glutamate signaling in addiction.

There is an important role of steroidal hormones in mediating immune functions, including 17β-estradiol (E2), its weaker estrogen metabolites such as estrone (Schmidt et al., 2009), as well as the steroidal hormone progesterone [217]. The metabolism of these steroidal hormones is complex, as cholesterol is the precursor for pregnenolone which converts to progesterone, which can then be converted to androstenedione, which is converted to testosterone, and then E2. It is therefore plausible that different steroidal hormones as well as their metabolites play key roles in neuroimmune processes involved in addiction. As noted above, testosterone is converted to E2 in both males and females [191]. Thus, it is biologically feasible that steroidal hormones also interact with neuroimmune signaling and glutamate homeostasis in critical ways relevant to addiction in males. However, to the notion that this may be sex-specific, one study found that extracellular striatal dopamine not only varies by estrous cycle phase in rats, but that gonadectomy in females (via ovariectomy) but not males significantly decreased striatal dopamine levels compared to intact counterparts [334]. Although this study focused on dopamine release and not glutamate or neuroimmune systems, these results indicate that the reward pathway is likely differentially regulated by steroidal hormones in males and females and supports further research on sex differences in this area. Given the complex shifting steroidal hormone milieu during reproductive cycling in females, as well as the metabolic relationships between steroidal hormones, much research is needed to fully characterize and understand how they interact with the NF-κB pathway and glutamate signaling in addiction. It is also important to note that males and females undergo vastly different reproductive system transitions during normal aging, whereby females undergo a precipitous loss of steroidal hormones during menopause, and males undergo a slow decline in hormones, termed andropause [24, 108]. As well, precipitous loss of E2 during menopause may increase vulnerability to pro-inflammatory signaling, which may be further exacerbated due to factors which have antiestrogenic properties such as smoking [225, 304]. Thus, aging processes should also be assessed in a sex-specific fashion.

Due to the recent push by the National Institutes of Health to incorporate both sexes into scientific research, as well as the focus by the National Institute on Drug Abuse to focus specifically on issues relevant to SUDs in women, sex differences in neurobiology and reproductive life cycle that could interact with drugs of abuse have become more frequently investigated. As a result, important findings have emerged in the study of aging and SUDs in women, an area of research that has been largely ignored. For example, the presence of menopausal symptoms is associated with decreased rates of smoking abstinence [62]. Below we describe how estrogens interact with neuroimmune processes, which may be important in mediating motivated drug use in addiction**.**

Estrogens, primarily E2, bind to estrogen receptors (ER)-α and ER-β. ER-α and ER-β are expressed in a variety of cell types [204] and can directly regulate expression of numerous genes and alter transcription without directly binding to DNA by associating with other transcription factors [260]. Through these powerful mechanisms, estrogens can greatly impact cellular function. Importantly, estrogen can be neuroprotective. During transitional menopause, a precipitous loss of estrogen occurs and is associated with neurodegenerative disorders as well as neuroinflammatory diseases and vascular wall degeneration [220]. Thus, the loss of estrogen during menopause may lead to neuroinflammatory processes that exacerbate disease, specifically in women.

Estrogens can interact with NF-κB directly (Fig. 1). They primarily repress monocyte and macrophage functions [124], and E2 specifically inhibits NF-κB signaling through inhibition of IL-6 and TNFα [58, 136]. Further, a significant amount of evidence suggests that estrogen inhibits microglial activation [220, 322, 323]. ERs are transcription factors that mediate responses to estrogen and are essential for various biological processes including cardiovascular, reproductive, and nervous systems [150]. ERs can inhibit NF-κB activity via various mechanisms including inhibition of IKK activity, inhibition of the degradation of IκB, blocking binding of DNA by NF-κB, binding coactivators and competing with NF-κB for coactivator binding, or binding directly to DNA-bound NF-κB to inhibit transcriptional activation induced by NF-κB. Activation of ER-α inhibits NF-κB activity in an estrogen-dependent manner, at nanomolar concentrations of estrogen [292, 319]. Further, ER expression is rapidly and transiently decreased following estradiol-induced activation (termed “receptor recycling” [38, 160]). This rapid alteration in ER expression may impact neuroimmune signaling through interactions with NF-κB, leading to the possibility that induction of NF-κB activation due to drug seeking may be decreased during phases of the menstrual cycle in which E2 levels rise. The anti-inflammatory effect of E2 is further supported by the finding that E2-induced activation of G protein-coupled receptor 30 (GPR30), an ER highly expressed in the brain, inhibited the TLR4/NF-kB pathway, relieved microglial activation, and reduced TNFα levels after ischemic injury [342], The presence of GPR30 in the striatum [6] supports the need for further research of its role in addiction processes.

Growing evidence suggests that the neuroprotective effects of E2 on neuronal health occur through the ability of E2 to modulate synaptic glutamate levels [243]. Evidence shows that blood glutamate levels vary as a function of menstrual cycle phase in women, where glutamate levels decline as E2 and progesterone increase [348]. Further, E2 increases GLT-1 expression in astroglia [180], which may be protective from the long-term reductions in glutamate uptake induced by drugs of abuse. Together, these results indicate that glutamate levels decrease in preparation for ovulation as a potentially direct consequence of rising E2 levels. However, it may be possible that the decrease in glutamate from rising E2 levels may exacerbate drug use vulnerability, given that low constitutive glutamate levels occur following withdrawal from drugs of abuse [20] and are associated with potentiated synapses within the NA [110]. Further research is needed to unravel the complex interactions between hormones, neural circuitry, and addiction in females.

In support of a critical role for the shifting ovarian hormone milieu in regulating drug motivation, studies have shown that drug self-administration varies as a function of estrous cycle phase in rodents [196]. As well, clinical studies have found that women are typically more vulnerable to SUDs [138, 139], and women have more difficulty maintaining long-term smoking cessation than men [249, 250, 329]. Interestingly, menstrual cycle phase in women can affect cigarette craving and relapse following periods of abstinence [5, 54, 95], and this may be due to the shifting ovarian hormone milieu. Specifically, increases in E2 and progesterone are associated with addiction vulnerability and resilience, respectively [8, 285–288]. Given that E2 can be neuroprotective, these are counterintuitive results. Thus, additional research is needed to understand how the changing ovarian hormone milieu with menstrual cycle may differentially impact drug-seeking motivation and glutamate plasticity in addiction.

## Interactions between microglia and astroglia

In the section “Developing an understanding of the interactions between neuroimmune signaling and glutamate in addiction”, we described ways in which microglia and astroglia interact with glutamate signaling particularly in the context of addiction. However, microglia and astroglia communicate directly as well, which influences neuronal function and survival. In this section, we will first describe the important early functions of microglia and astroglia and will then describe their interactions. Both microglia and astroglia are critical in shaping the brain during early life development, as microglia development and maturation is synchronized with neurogenesis and are important in synaptic pruning [265], and astroglia contribute to the formation of neural circuits [55].

Astroglia form complex networks that are ubiquitously found in all brain regions, spinal cord, and all neuronal layers. They are connected by gap junctions and form long processes with end feet that communicate with blood vessels and also ensheathe synapses [201]. Astroglial networks are vast, and these cells organize communication pathways, structural architecture, and plasticity of the brain [231]. In addition to glutamate uptake (see section “Developing an understanding of the interactions between neuroimmune signaling and glutamate in addiction” above), astroglia are also critical in transferring glutamate back to neurons, supply energy substrates to neurons, store glucose, regulate pH in the brain microenvironment, serve a neuroprotective role against oxidative stress, and play a role in neuroimmune responses (for a review on astroglial functions, see [27]). As such, astroglia play a critical role in maintaining brain homeostasis and have been heavily implicated in addiction (see [151, 270]).

Microglia are exceptionally morphologically plastic and dynamic [12], whereby microglial processes rapidly traverse the parenchyma at a rate of 1–3 μm/min [232]. The reason for this cephalopod-like activity is to maintain sampling of the interstitial fluid at a relatively constant rate, and to both respond to pathogens and influence synaptic transmission with the release of neuromodulators [163]. Strikingly, morphological properties of microglial cells are thought to align with their functional cellular activity states [32, 158, 223]. Resting microglia are often characterized by numerous thin and elongated processes. Conversely, neuroimmune activation can transition microglia into a “reactive” state, characterized by an enlarged soma and a simplified or reduced process field. Once activated, microglia express secretory analogs that act to defend the central nervous system from environmental insults [40, 53]. It is important to note that a binary categorization of microglia has been used in the past to differentiate activation states of microglia, but this does not accurately reflect the array of microglial activation states. Specifically, M1 pro-inflammatory phenotype and M2 anti-inflammatory phenotype were defined by their differential expression of receptors thought to be involved in pro- or anti-inflammatory roles. Recently, however, a primed phenotype has been proposed in which microglia are not fully activated in cases of incomplete injury or repetitive mild injury, and hyper-activated microglia are hypothesized to result from chronic inflammation [173]. While these categories of activation states are ubiquitously observed in neuroinflammatory dysfunction, the complex molecular profile of microglia across a spectrum of activation has attenuated their utility. Within the field of addiction, markers associated with both M1 and M2 stages were shown after 4-day binge alcohol exposure in multiple studies [246, 247]. These results support invalidation of this binary classification system and support a more nuanced and rigorous approach to characterizing microglial activation states.

As described above, microglia and astroglia are dynamic and constantly moving cells. These cells form quad-partite synapses with glutamatergic neurons [198] and contribute significantly to brain homeostasis. Further, microglia and astroglia are part of the innate immune system, and crosstalk between these cells is necessary for astroglia to support survival and function of neurons after injury (e.g., see [123]). Microglia and astroglia are intimately involved in neuroimmune signaling and communicate with each other through various signaling molecules [201]. Next, we will therefore describe these interactions and how they may play a critical role in driving aberrant drug-seeking behavior.

Microglia and astroglia communicate and contribute to inflammatory responses in the brain. Studies have shown that they do this through signaling molecules such as glutathione S-transferases GSTM1 and GSTT2 [157], as well as secreted mediators such as the pro-inflammatory cytokines TNFα, interleukin (IL)-1β, IL-6, IL-18, and IL-10 after tissue injury [98]. These cytokines are produced when dyshomeostasis is detected by microglia, astroglia, progenitor cells, oligodendrocytes, and neurons. Although it is well documented that microglia release pro-inflammatory cytokines such as TNFα (for a review, see [240]), activated astroglia also release pro-inflammatory cytokines such as IL-1β [337] and TNFα [44, 60, 96]. Through interactions with tumor necrosis factor receptor 1 (TNFR1), TNFα initiates intracellular signaling cascades leading to the generation of the prostaglandin E_2_ [33, 34], which then leads to elevated intracellular Ca^2+^ and glutamate exocytosis. The resulting excessive glutamate then cannot be cleared from the extracellular space due to the abovementioned TNFα-induced reduction in astroglial glutamate uptake [101, 313, 346].

Glial cells are known to regulate and control the function of each other, as well as their migration and reactions, especially in the context of disease. This is important for astroglia to support neuronal survival following an injury. Further, microglia appear to be a first-line defense against injury given their rapid recruitment to sites of damage and phagocytosis of dead cells [123]. A specific example of astroglia-microglia communication in disease is found in obesity-induced hypothalamic inflammation. This disease state is associated with direct binding of astroglial 4-1BB (which is a member of the TNF receptor superfamily) to its ligand which is expressed on microglia (4-1BBL; see [166]). The result of this binding is release of pro-inflammatory cytokines such as TNFα and IL-6. Thus, there are direct astroglia-microglia interactions that are involved in responses to disease, and this supports the tenet that these interactions may impact glutamate homeostasis which may be critical in driving drug-seeking motivation.

### Quantifying interactions between microglia and astroglia with structural precision

This section is focused on new technology allows for more intricate connections to be made between structure and function of non-neuronal cells. This is critical to highlight here given the movement in the field away from the binary classification system of microglia. Advances in technology could therefore provide more detailed information regarding structural signatures that reflect these nuanced activation states.

**Fig. 2:**
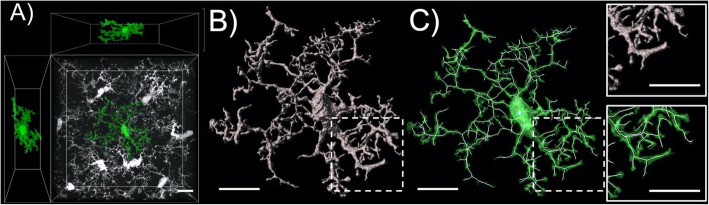
High-resolution 3-D image of an isolated Iba-1-positive microglia with orthogonal views. **a** Iba-1 labeling and modern confocal microscopy can be reliable used to label and image microglial syncytia (white)**.** Within these syncytia, individual microglia (green) can be digitally isolated from neighboring cells and subsequently analyzed. **b** Here a space filling 3D render of the isolated microglia is shown (grey). This type of digital analyses can be used to obtain data for general physical parameters (surface area and volume). **c** Here the microglial cell of interest is shown (green) overlaid with a skeletonization (white). This type of analysis can be used to obtain structural characteristics (Sholl intersections, branch number, and branch order). Hashed boxes depict locations of inset panels. Scale bar depicts 10 μm

As glial responses have become prevalent indicators of neuroimmune signaling in many different contexts, including addiction, the methods used to quantify glial morphology have been increasingly developed. The remarkable structure of both microglia and astroglia, as well as their clear structure-function relationship, has contributed significantly to the propensity for these cells to be studied with microscopy (Fig. 2). As mentioned in prior sections, the canonical binary classification of microglial activation states is no longer considered a valid metric by which microglia should be distinguished. New technology allows for more intricate connections to be made between structure and function to be made, and as such, advances in technology could provide more detailed information regarding structural signatures that reflect these nuanced activation states. Below we describe advances in technology that make it possible to characterize more nuanced microglial activation states.

Early information on the structural diversity of astroglia and microglia and an understanding of how these cells change morphologically following environmental insult were typically obtained using standard widefield microscopy procedures. In this early work, morphological analyses were often conducted with high-contrast immunohistochemical labeling utilizing canonical markers for each cell type; glial fibrillary acidic protein (GFAP) for astroglia and ionized calcium-binding adaptor molecule 1 (Iba1) for microglia. At first, these data sets were often acquired at relatively low magnification using a stereological approach for cell counting. In this design, large numbers of cells were simultaneously imaged, with primary early outputs being cell number and the relative intensity of the label used. Intensity of the signal was a particularly common early output variable as the signal brightness for GFAP was used as an index of reactivity in the case of astroglia [176, 284] and signal brightness of Iba1 and activation state in the case of microglia [43, 97, 142]. When specifics about the morphological aspects of microglia and astroglia were initially investigated, multiple optical sections within each data set were not always obtained, and when this type of sampling was performed, sections were often collapsed into flattened 2D images for the final analysis of cellular structure, significantly restricting the span of processes that can be measured [1, 129, 338]. While these studies provided vital information, advancements in microscopy, 3D digital rendering, and analysis techniques have advanced our ability to observe and analyze non-neuronal cells.

More recently, high-magnification 3D imaging and analysis of morphometrics have been performed on microglia and astroglia, often utilizing new techniques for genetic and viral vector-based labeling [75, 175, 271, 277, 308–310, 314]. The usage of modern genetic and viral labeling in conjunction with updated confocal microscopy methods has advanced morphological analysis of non-neuronal cells, improving upon earlier strategies which use incomplete or inconsistent cellular labeling, limited optical sampling, and lower resolution. Ultimately, the application of modern microscopy and digital rendering and analysis techniques will continue to improve our understanding of these structurally dynamic cell types [10, 170, 222, 271]. Recent advancements in the resolution of confocal microscopy have become achievable through technical improvements in microscope detectors; these technical advances have been combined with enhanced deconvolution algorithms that more precisely account for light scattering. These techniques and expanding technologies allow for super-resolution light microscopy [71, 137, 321]. Accordingly, the fidelity of the digital reconstruction of non-neuronal cell structure acquired with these techniques has improved dramatically over the last decade [268]. However, these methods are just beginning to be harnessed for the morphological analysis of astroglia and microglia at the single-cell level with sub-micron precision, so further expansion in analysis of non-neuronal cell types with this technology is necessary [143, 264, 275].

Given the clear structure-function relationship described in the section above, microglia are often analyzed optically to assess their activation state. As described above, increased soma size and reduced branching patterns commonly act as structural biomarkers for activation [72, 94, 222]. While the structural properties of microglia are commonly used to classify them into either an activated or a resting state, we describe above that a binary two-state categorization is overly simplistic (i.e., M1 and M2), as modern perspectives indicate the presence of a continuum of unique activation states [232]. As a result, parameters like cell area, cell volume, process length, branch points, Sholl intersections, and the territory occupied by microglial processes are all commonly used to interpret and understand how changes in morphological characteristics relate to microglial activation [94, 132, 222]. Microglial complexity has also been quantitatively measured using fractal dimension, which is expressed as the ratio of increasing detail to increased magnification seen in microscopy [147]. More recent examinations include levels of IL-1β cluster of differentiation 68 (CD68), or TNFα expression within individual microglial cells as additional metrics for understanding their relative activation states [93, 161, 184, 344].

Output variables and procedures to analyze microglial morphological properties have been more firmly established than what has been the case for their astroglial counterparts. There has been a considerable amount of difficulty in investigating astroglial structure, as astroglial branching processes are relatively small and highly ramified [130]. Accordingly, the usage of older light microscopy methods often makes these processes difficult to isolate and resolve. In addition, while GFAP is a useful marker for investigating astroglia, a well know problem is that even the thinnest GFAP branches do not extend into very fine peri-synaptic processes of astroglia, thus measures of density are limited in their quantification of astroglial morphology [125]. As described above, for the highest fidelity confocal images of the astroglial plasma membrane, dye filling, genetic labeling, or viral vectors are needed. The work of the Khakh lab among others has been seminal in this regard, with the design and implementation of reliable membrane-targeted cell type-specific reporter and Ca^2+^ indicator constructs. These vectors reveal a striking astroglial cellular architecture that extends well beyond the GFAP arbor [164, 273, 274]. Notably, these constructs reveal an elegant nebulous cellular structure that is far more complex than what is revealed by GFAP immunohistochemistry and also much more complete than what can be observed with conventional cytosolic GFAP-promoter driven reporter constructs. The Khakh group continues to innovate in this area, constructing and implementing improved tools to study astroglia and their interaction with neurons [14, 339, 340]. It is also important to note that even the most advanced light microscopy cannot be used to completely visualize the finest astroglial processes, which are estimated to be approximately as thin as 30 nm at the tips [30, 130, 164]. Thus, electron microscopy (EM) has been extremely valuable for the analysis of the organization of non-neuronal membrane processes [100, 130]. While 3D serial EM is very labor intensive, this technique has also been used to construct breathtaking reconstructions of the complex processes of astroglia and microglia and their interactions with neurons [30, 41, 51, 195, 233, 263].

## Targeting neuroimmune and glutamate signaling for pharmacotherapeutic treatment of substance use disorders

In this section, we will highlight neuroimmune-glutamate mechanisms as potential targets for pharmacotherapies to treat SUDs. We will detail compounds that are currently being examined clinically and/or preclinically which may restore glutamate homeostasis, potentially through interactions with neuroimmune signaling. We will also highlight neuroimmune dysregulations that have been found clinically and/or preclinically, which may be important for future pharmacotherapeutic targeting.

Neuropsychiatric diseases, including SUDs, are influenced by interactions between neurons and glia that impact glutamate homeostasis. As discussed above, disruptions in glutamate homeostasis and glutamate transporter function (specifically, GLT-1) in brain reward circuits during psychostimulant exposure contribute to reinforcement and relapse. Putative therapeutics that enhance the expression of GLT-1 (e.g., ceftriaxone, *N*-acetylcysteine (NAC), riluzole) decrease drug-seeking behaviors in preclinical assays [168, 262, 326]. NAC is the most commonly studied glutamate compound in clinical trials [116, 197, 202], yet has shown checkered clinical success (e.g., [178]). Emerging evidence suggests that pharmacological agents acting upstream of glutamate transport systems (e.g., GLT-1 and system Xc) to influence broader aspects of glial function also disrupt drug reinstatement and relapse. One of the most studied agents is propentofylline (PPF), which is a pharmacologically diverse methylxantine derivative that acts through multiple mechanisms, including enhancement of GLT-1 transporter expression, inhibition of phosphodiesterase, and enhancement of adenosine uptake [302, 305]. Methylxanthines are approved by the Food and Drug Administration (FDA) to treat asthma and peripheral vascular disease, including intermittent claudication [86, 226], and are thus highly amenable for repurposing to other therapeutic indications. Evidence indicates that PPF, when administered systemically (but not acutely) reduces relapse to cocaine seeking that is induced by both cues and exposure to cocaine itself [254]. Moreover, the efficacy of PPF is cocaine-specific and does not extend to effects on sucrose seeking. Interestingly, the suppression of cocaine relapse by PPF was linked to changes in glutamate transport function, as the efficacy of PPF was dependent on the restoration of GLT-1 expression within the NA. Although the mechanism by which PPF reduces relapse to cocaine seeking is not entirely understood, it seems reasonable that normalization of glutamate homeostasis through restoration of astroglia-medicated clearance of extracellular glutamate is a contributing factor [99, 228]. Other potential explanations for how PPF reduces cocaine seeking include inhibition of phosphodiesterase, which reduces cocaine sensitization and reinforcement [188, 213, 267, 345], and interactions with adenosine A_1_ or A_2_ receptors, which influence behavioral responses to cocaine [47, 236, 315]. The efficacy of PPF also extends to other drugs of abuse, as systemic treatment with PPF blocks rewarding effects of methamphetamine and morphine in conditioned place preference assays [229].

As mentioned above, activation of adenosine receptors may influence drug-seeking behavior, and likely does so through the regulation of glutamatergic signaling. For example, acute ethanol exposure elevates extracellular adenosine levels by selective inhibition of the type 1 equilibrative nucleoside transporter (ENT1), and it was found that genetic deletion of ENT1 resulted in reduced expression of EAAT2 [257]. Overexpression of adenosine 2A (A_2A_) receptors via a neuron-specific enolase promoter in rats receiving nicotine treatment resulted in a rise in glutamate levels, whereas the opposite effect was found in wildtype nicotine-treated animals [146]. Future studies should examine small molecule compounds that upregulate ENT1 and A_2A_ as indirect mechanisms that consequently restore EAAT2 expression and rescue glutamate homeostasis within the context of addiction. Furthermore, sex differences in these outcomes are critical for enhancing translatable findings.

An increasing body of evidence suggests that purinergic systems, through broad regulators of glial cell physiology and possible effects on glutamate homeostasis, influence SUDs. In particular, purinergic P2X7 receptors, which are expressed by astroglia and microglia and activated by extracellular ATP, stand out as key elements of the purinergic system that are linked to physiological underpinnings of drug reward, reinforcement, and relapse. Activation of P2X7 receptors causes microglia to become activated and release endogenous substrates, including glutamate, dopamine, pro-inflammatory cytokines (e.g., IL-1β, IL-6 and TNFα), and reactive oxygen species, that facilitate psychostimulant reward and reinforcement [91, 92, 127, 245], suggesting a link between the pro-inflammatory actions of P2X7 and CNS diseases. Recent evidence indicates that P2X7 receptor blockade with the competitive, reversible P2X7 antagonist A438079 inhibits facilitation of intracranial self-stimulation (ICSS) by the psychostimulant methylenedioxypyrovalerone (MDPV), a “bath salt” synthetic cathinone with a mechanism of action similar to cocaine but with enhanced potency in blocking dopamine transporters [26, 102]. The ability of A438079 to reduce psychostimulant reward enhancement in an ICSS assay is most likely due to reduction of P2X7 receptor activity, as A438079 (IC50 = 100 and 300 nm at rat and human P2X7 receptors, respectively) lacks significant activity at other purinergic type 2 (P_2_) receptors (IC50 > 10 μM), shows negligible activity at non-purinergic receptors and ion channels, and penetrates the brain [82]. Cellular experiments also show that P2X7 mRNA levels and P2X7 receptor protein expression within the NA are increased by MDPV, further supporting a role for P2X7 receptors in psychostimulant reward enhancement [102]. However, elevated P2X7R protein was also found in spinal microglia of morphine-dependent rats, demonstrating its involvement in reward-seeking behavior induced by an array of drug types [50]. Because both P2X7 receptor activation and chronic psychostimulant exposure induce neuroinflammation, which facilitates psychostimulant dependence, it is plausible that psychostimulants such as MDPV, cocaine, and methamphetamine induce upregulation of P2X7 receptors leading to downstream release of pro-inflammatory cytokines (e.g., IL-1, IL-6 and TNFα) that contribute to abuse liability [13, 174, 298]. It is also worth mentioning that P2X7 receptor antagonism or genetic deletion reduces hyperactivity induced by amphetamines [35, 69, 118]. It has also been found that modulation of the P2X4 receptor mediates the enhancement of microglial migration by morphine [135], and alcohol exposure leads to upregulation of P2X4 receptors in embryonic stem cell-derived microglia cells [112]. There is limited study of the involvement of other purinergic receptors in behavior underlying SUDs, and while P2Y12 expression was found to be lower in murine female microglia than in male, sex differences in purinergic receptors have not been evaluated in the context of addiction.

Although central functions of chemokines were originally thought to be limited to chemotaxis and neuroinflammation, a growing body of evidence suggests that chemokine systems influence physiological circuits, including dopaminergic and perhaps glutamatergic, which underlie drug addiction [2, 70]. The CXCL12/CXCR4 chemokine ligand/receptor pair is one particular interest. CXCL12 (e.g., stromal cell-derived factor one alpha (SDF-1α)) is one of the few chemokines found in the brain and is secreted by neuronal and non-neuronal populations [120, 295]. CXCL12 binds to and activates at least two receptors, CXCR4 and CXCR7, with the former being the major receptor for CXCL12 in the brain that is expressed by neurons, astroglia, and microglia [19, 39]. CXCR4 receptor immunoreactivity is expressed by dopamine neurons in the substantia nigra [23] and GABAergic MSNs in the lateral shell of the NA [316]. The FDA has approved a CXCR4 antagonist called AMD3100 (Plerixafor) that is available to investigate receptor function and displays selectivity for CXCR4 against other chemokine receptors (e.g*.*, CXCR1 through CXCR3, or CCR1 through CCR9 [332]).

In the context of cocaine use disorder, plasma levels of CXCL12 are decreased in human cocaine abusers during withdrawal and elevated in mice following acute cocaine exposure [9]. Notably, CXCL12 is one of only two chemokines (CXCL12 and CX3CL1) altered in the plasma of cocaine abusers during abstinence and the only chemokine found to be positively correlated with the history of pathological cocaine use and severity of dependence [9]. CXCL12 has been found to have a variety of affects in different brain regions. For example, when administered into the lateral ventricles or ventral tegmental area (VTA), CXCL12 enhances locomotor activation produced by cocaine [317]. CXCL12 injected into the substantia nigra enhances extracellular dopamine in the dorsal striatum in a CXCR4 receptor-dependent manner [120, 279]. More recently, CXCR4 antagonism by AMD3100 was shown to reduce cocaine conditioned place preference and locomotor activation [165], and similar effects were observed for AMD3100 against MDPV [239]. At the cellular level, both cocaine and MDPV enhance CXCL12 gene expression in the mesolimbic circuit [165, 239]. Although the mechanisms underlying the efficacy of AMD3100 against psychostimulant reward and locomotor activation are unclear, a downstream reduction of mesolimbic dopamine transmission through CXCR4 receptor blockade is a plausible explanation based on the current literature. Given that CXCR4 receptor activation reduces glutamate release in mouse cerebellar slices [251], interactions between CXCR4 and glutamate systems in mesocorticolimbic circuits may also play a role, and future studies should investigate how CXCR4-glutamate crosstalk impacts psychostimulant reinforcement and relapse.

Studies have shown that the receptor CX3CR1 is involved in cocaine use disorder. Communication between CX3CR1 and its ligand, fractalkine (CX3CL1), mediates neuroprotection and promotes microglial activation [252]. Further, this interaction between fractalkine and CX3CR1 has been implicated in cocaine dependence in the mouse hippocampus following social defeat (SD [218];). After exposure to cocaine-induced CPP, WT-SD mice showed an increase in the p-p65/p65 NF-κB ratio and pCREB/CREB, while CX3CR1-KO-SD mice exhibited opposite changes [218]. These findings suggest a role of CX3CR1 in the activation of transcription factors that modulate the development of CPP, though further research is needed to establish its role in other drug-seeking behaviors. While no change in glutamatergic receptor subunit protein expression was found in CX3CR1-KO mice, another study discovered a low AMPA/NMDA ratio in CX3CR1-KO mice during development, leaving open the question of involvement of CX3CR1 in glutamate signaling. Further, sex differences have been discovered in the modulation of inflammatory response by CX3CR1 whereby female CX3CR1-KO mice showed WT “male-like” microglial activation in response to diet-induced inflammation [83]. Taken together, these studies lay the groundwork for further research on sex differences in SUD-induced neuroinflammation and may warrant sex-specific lines of research for pharmacotherapeutic development to treat SUDs.

## Conclusions

Here we propose that neuroimmune signaling impacts glutamate homeostasis in the neurobiological processes underlying drug addiction. The role of neuroimmune processes in SUDs is not well understood and is an exciting and novel area of research. Notably, tools to examine the role of pro-inflammatory signals and cellular morphology with high resolution are currently being developed, which will allow for unprecedented understanding of how neuroimmune signaling impacts glutamate plasticity and drug-seeking motivation. There is also a plethora of evidence for sex differences in neuroimmune mechanisms involved in glutamate homeostasis in addiction. Further, small molecule pharmacotherapies that target the glutamate system have been studied at both the preclinical and clinical levels which may impact neuroimmune signaling, such as NAC, propentofylline, or β-lactam antibiotics such as ceftriaxone. However, additional pharmacotherapeutic development is needed given the checkered clinical efficacy of some of these glutamatergic compounds (e.g., NAC [107, 178]). Taken together, we propose a neuroimmune-glutamate circuit that is critical in driving use of drugs of abuse, which may be sex-specific and impacted by steroid hormones.

## Data Availability

All data and materials are available upon request.

## Abbreviations

A_2A_: Adenosine 2A
AMD3100: Plerixafor
AMPA: α-Amino-3-hydroxy-5-methyl-4-isoxazolepropionic acid
BBB: Blood-brain barrier
BDNF: Brain-derived neurotrophic factor
CCP: Conditioned place preference
CD68: Cluster of differentiation 68
CNS: Central nervous system
CX3CR1: C-X3-motif ligand 1 (fractalkine)
Cx3cr1: CX3C chemokine receptor 1
E2: 17β-estradiol
EAAT2: Excitatory Amino Acid Transporter-2
ED_50_: Half-maximal antinociceptive dose
EM: Electron microscopy
ENT1: Type 1 equilibrative nucleoside transporter
ER-α: Estrogen receptor-α
ER-β: Estrogen receptor–β
GDNF: Glial cell line-derived neurotrophic factor
GFAP: Glial fibrillary acidic protein
GLT-1: Glial glutamate transporter-1
GPR30: G protein-coupled receptor 30
Iba1: Ionized calcium-binding adaptor molecule 1
ICSS: Intracranial self-stimulation
IKK: Interferon-gamma IκB kinase
IL-1: Interleukin-1
IL-1β: Interleukin-1 beta
IL-6: Interleukin-6
IL-8: Interleukin-8
IL-37: Interleukin-37
JNK: c-Jun N-terminal kinase
LPS: Lipopolysaccharide
MD-2: Myeloid differentiation factor 2
MDPV: Methylenedioxypyrovalerone
mGluRs: Metabotropic glutamate receptors
miR: microRNA
MMP9: Matrix metalloproteinase 9
MMPs: Metalloproteinases
mPFC: Medial prefrontal cortex
MSNs: Medium spiny neurons
mTOR: Mammalian target of rapamycin
NAC: *N*-acetylcysteine
NAcore: Nucleus accumbens core
NA: Nucleus accumbens
NF-κB: Nuclear factor-kappa B
NMDA: *N*-Methyl-d-aspartic acid
nNOS: Neuronal nitric oxide synthase
NO: Nitric oxide
P_2_: Purinergic type 2
PPF: Propentofylline
PPRs: Pattern recognition receptors
SD: Social defeat
SDF-1α: Stromal cell-derived factor one α
SUDs: Substance use disorders
TLRs: Toll-like receptors
TNFα: Tumor necrosis factor α
TNFR1: Tumor necrosis factor receptor 1
VTA: Ventral tegmental area
WT: Wildtype
xCT: Cystine-glutamate antiporter

## References

[1] Abdolhoseini M, Kluge MG, Walker FR, Johnson SJ (2019). Segmentation, tracing, and quantification of microglial cells from 3D image stacks. Sci Rep.

[2] Adler MW, Geller EB, Chen X, Rogers TJ (2006). Viewing chemokines as a third major system of communication in the brain. AAPS J.

[3] Alam S, Laughton DL, Walding A, Wolstenholme AJ (2006). Human peripheral blood mononuclear cells express GABAA receptor subunits. Mol Immunol.

[4] Albensi BC, Mattson MP (2000). Evidence for the involvement of TNF and NF-kappaB in hippocampal synaptic plasticity. Synapse.

[5] Allen SS, Bade T, Center B, Finstad D, Hatsukami D (2008). Menstrual phase effects on smoking relapse. Addiction.

[6] Almey A, Milner TA, Brake WG (2016). Estrogen receptor alpha and G-protein coupled estrogen receptor 1 are localized to GABAergic neurons in the dorsal striatum. Neurosci Lett.

[7] Ang E, Chen J, Zagouras P, Magna H, Holland J, Schaeffer E, Nestler EJ (2001). Induction of nuclear factor-kappaB in nucleus accumbens by chronic cocaine administration. J Neurochem.

[8] Anker JJ, Larson EB, Gliddon LA, Carroll ME (2007). Effects of progesterone on the reinstatement of cocaine-seeking behavior in female rats. Exp Clin Psychopharmacol.

[9] Araos P, Pedraz M, Serrano A, Lucena M, Barrios V, Garcia-Marchena N, Campos-Cloute R, Ruiz JJ, Romero P, Suarez J, Baixeras E, de la Torre R, Montesinos J, Guerri C, Rodriguez-Arias M, Minarro J, Martinez-Riera R, Torrens M, Chowen JA, Argente J, Mason BJ, Pavon FJ, Rodriguez de Fonseca F (2015). Plasma profile of pro-inflammatory cytokines and chemokines in cocaine users under outpatient treatment: influence of cocaine symptom severity and psychiatric co-morbidity. Addict Biol.

[10] Ardalan M, Rafati AH, Nyengaard JR, Wegener G (2017). Rapid antidepressant effect of ketamine correlates with astroglial plasticity in the hippocampus. Br J Pharmacol.

[11] Ataka K, Asakawa A, Nagaishi K, Kaimoto K, Sawada A, Hayakawa Y, Tatezawa R, Inui A, Fujimiya M (2013). Bone marrow-derived microglia infiltrate into the paraventricular nucleus of chronic psychological stress-loaded mice. PLoS One.

[12] Avignone E, Lepleux M, Angibaud J, Nagerl UV (2015). Altered morphological dynamics of activated microglia after induction of status epilepticus. J Neuroinflammation.

[13] Bachtell RK, Jones JD, Heinzerling KG, Beardsley PM, Comer SD (2017). Glial and neuroinflammatory targets for treating substance use disorders. Drug Alcohol Depend.

[14] Badia-Soteras A, Octeau JC, Verheijen MHG, Khakh BS (2020). Assessing neuron-astrocyte spatial interactions using the neuron-astrocyte proximity assay. Curr Protoc Neurosci.

[15] Bai J, Li Y, Li M, Tan S, Wu D (2020). IL-37 as a potential biotherapeutics of inflammatory diseases. Curr Drug Targets.

[16] Bai Y, Zhu Z, Gao Z, Kong Y (2014). TLR2 signaling directs NO-dependent MMP-9 induction in mouse microglia. Neurosci Lett.

[17] Bailey J, Ma D, Szumlinski KK (2012). Rapamycin attenuates the expression of cocaine-induced place preference and behavioral sensitization. Addict Biol.

[18] Bailey SL, Carpentier PA, McMahon EJ, Begolka WS, Miller SD (2006). Innate and adaptive immune responses of the central nervous system. Crit Rev Immunol.

[19] Bajetto A, Bonavia R, Barbero S, Florio T, Schettini G (2001). Chemokines and their receptors in the central nervous system. Front Neuroendocrinol.

[20] Baker DA, McFarland K, Lake RW, Shen H, Tang XC, Toda S, Kalivas PW (2003). Neuroadaptations in cystine-glutamate exchange underlie cocaine relapse. Nat Neurosci.

[21] Baker DA, Shen H, Kalivas PW (2002). Cystine/glutamate exchange serves as the source for extracellular glutamate: modifications by repeated cocaine administration. Amino Acids.

[22] Balan I, Warnock KT, Puche A, Gondre-Lewis MC, June H, Aurelian L. The GABAA receptor alpha2 subunit activates a neuronal TLR4 signal in the ventral tegmental area that regulates alcohol and nicotine abuse. Brain Sci. 2018;8. 10.3390/brainsci8040072 PMC5924408 29690521

[23] Banisadr G, Fontanges P, Haour F, Kitabgi P, Rostene W, Melik Parsadaniantz S (2002). Neuroanatomical distribution of CXCR4 in adult rat brain and its localization in cholinergic and dopaminergic neurons. Eur J Neurosci.

[24] Basaria S (2013). Reproductive aging in men. Endocrinol Metab Clin N Am.

[25] Batchelor PE, Liberatore GT, Wong JY, Porritt MJ, Frerichs F, Donnan GA, Howells DW (1999). Activated macrophages and microglia induce dopaminergic sprouting in the injured striatum and express brain-derived neurotrophic factor and glial cell line-derived neurotrophic factor. J Neurosci.

[26] Baumann MH, Partilla JS, Lehner KR, Thorndike EB, Hoffman AF, Holy M, Rothman RB, Goldberg SR, Lupica CR, Sitte HH, Brandt SD, Tella SR, Cozzi NV, Schindler CW (2013). Powerful cocaine-like actions of 3,4-methylenedioxypyrovalerone (MDPV), a principal constituent of psychoactive 'bath salts' products. Neuropsychopharmacology.

[27] Bélanger M (2009). The role of astroglia in neuroprotection. Dialogues Clin Neurosci.

[28] Bennett FC, Bennett ML, Yaqoob F, Mulinyawe SB, Grant GA, Hayden GM (2018). A combination of ontogeny and CNS environment establishes microglial identity. Neuron.

[29] Berglind WJ, Whitfield TW, LaLumiere RT, Kalivas PW, McGinty JF (2009). A single intra-PFC infusion of BDNF prevents cocaine-induced alterations in extracellular glutamate within the nucleus accumbens. J Neurosci.

[30] Bernardinelli Y, Muller D, Nikonenko I (2014). Astrocyte-synapse structural plasticity. Neural Plast.

[31] Bessis A, Bechade C, Bernard D, Roumier A (2007). Microglial control of neuronal death and synaptic properties. Glia.

[32] Beynon SB, Walker FR (2012). Microglial activation in the injured and healthy brain: what are we really talking about? Practical and theoretical issues associated with the measurement of changes in microglial morphology. Neuroscience.

[33] Bezzi P, Carmignoto G, Pasti L, Vesce S, Rossi D, Rizzini BL, Pozzan T, Volterra A (1998). Prostaglandins stimulate calcium-dependent glutamate release in astrocytes. Nature.

[34] Bezzi P, Domercq M, Brambilla L, Galli R, Schols D, De Clercq E, Vescovi A, Bagetta G, Kollias G, Meldolesi J, Volterra A (2001). CXCR4-activated astrocyte glutamate release via TNFalpha: amplification by microglia triggers neurotoxicity. Nat Neurosci.

[35] Bhattacharya A, Wang Q, Ao H, Shoblock JR, Lord B, Aluisio L, Fraser I, Nepomuceno D, Neff RA, Welty N, Lovenberg TW, Bonaventure P, Wickenden AD, Letavic MA (2013). Pharmacological characterization of a novel centrally permeable P2X7 receptor antagonist: JNJ-47965567. Br J Pharmacol.

[36] Biber K, Neumann H, Inoue K, Boddeke HW (2007). Neuronal 'On' and 'Off' signals control microglia. Trends Neurosci.

[37] Biber K, Laurie DJ, Berthele A, Sommer B, Tolle TR, Gebicke-Harter PJ, van C.D., Boddeke H.W. (1999). Expression and signaling of group I metabotropic glutamate receptors in astrocytes and microglia. J Neurochem.

[38] Blaustein JD (1993). Estrogen receptor immunoreactivity in rat brain: rapid effects of estradiol injection. Endocrinology.

[39] Bleul CC, Fuhlbrigge RC, Casasnovas JM, Aiuti A, Springer TA (1996). A highly efficacious lymphocyte chemoattractant, stromal cell-derived factor 1 (SDF-1). J Exp Med.

[40] Bohatschek M, Kloss CUA, Kalla R, Raivich G (2001). In vitro model of microglial deramification: Ramified microglia transform into amoeboid phagocytes following addition of brain cell membranes to microglia-astrocyte cocultures. J Neurosci Res.

[41] Bolasco G, Weinhard L, Boissonnet T, Neujahr R, Gross CT (2018). Three-dimensional nanostructure of an intact microglia cell. Front Neuroanat.

[42] Bonaiuto C, McDonald PP, Rossi F, Cassatella MA (1997). Activation of nuclear factor-kappa B by beta-amyloid peptides and interferon-gamma in murine microglia. J Neuroimmunol.

[43] Bosco A, Steele MR, Vetter ML (2011). Early microglia activation in a mouse model of chronic glaucoma. J Comp Neurol.

[44] Breder CD, Tsujimoto M, Terano Y, Scott DW, Saper CB (1993). Distribution and characterization of tumor necrosis factor-alpha-like immunoreactivity in the murine central nervous system. J Comp Neurol.

[45] Brevet M, Kojima H, Asakawa A, Atsuchi K, Ushikai M, Ataka K, Inui A, Kimura H, Sevestre H, Fujimiya M (2010). Chronic foot-shock stress potentiates the influx of bone marrow-derived microglia into hippocampus. J Neurosci Res.

[46] Brody AL, Hubert R, Enoki R, Garcia LY, Mamoun MS, Okita K, London ED, Nurmi EL, Seaman LC, Mandelkern MA (2017). Effect of cigarette smoking on a marker for neuroinflammation: a [(11)C]DAA1106 positron emission tomography study. Neuropsychopharmacology.

[47] Brown RM, Duncan JR, Stagnitti MR, Ledent C, Lawrence AJ (2012). mGlu5 and adenosine A2A receptor interactions regulate the conditioned effects of cocaine. Int J Neuropsychopharmacol.

[48] Brousse G, Arnaud B, Vorspan F, Richard D, Dissard A, Dubois M, Pic D, Geneste J, Xavier L, Authier N, Sapin V, Llorca PM, Chazeron ID, Minet-Quinard R, Schmidt J (2012). Alteration of glutamate/GABA balance during acute alcohol withdrawal in emergency department: a prospective analysis. Alcohol Alcohol.

[49] Buller KM (2003). Neuroimmune stress responses: reciprocal connections between the hypothalamus and the brainstem. Stress.

[50] Burma NE, Bonin RP, Leduc-Pessah H, Baimel C, Cairncross ZF, Mousseau M, Shankara JV, Stemkowski PL, Baimoukhametova D, Bains JS, Antle MC, Zamponi GW, Cahill CM, Borgland SL, De Koninck Y, Trang T (2017). Blocking microglial pannexin-1 channels alleviates morphine withdrawal in rodents. Nat Med.

[51] Cali C, Agus M, Kare K, Boges DJ, Lehvaslaiho H, Hadwiger M, Magistretti PJ (2019). 3D cellular reconstruction of cortical glia and parenchymal morphometric analysis from Serial Block-Face Electron Microscopy of juvenile rat. Prog Neurobiol.

[52] Calsolaro V, Edison P (2016). Neuroinflammation in Alzheimer’s disease: current evidence and future directions. Alzheimers Dement.

[53] Capriles N, Rodaros D, Sorge RE, Stewart J (2003). A role for the prefrontal cortex in stress- and cocaine-induced reinstatement of cocaine seeking in rats. Psychopharmacology.

[54] Carpenter MJ, Upadhyaya HP, LaRowe SD, Saladin ME, Brady KT (2006). Menstrual cycle phase effects on nicotine withdrawal and cigarette craving: a review. Nicotine Tob Res.

[55] Clarke LE, Barres BA (2013). Emerging roles of astrocytes in neural circuit development. Nat Rev Neurosci.

[56] Chen BT, Yau HJ, Hatch C, Kusumoto-Yoshida I, Cho SL, Hopf FW, Bonci A (2013). Rescuing cocaine-induced prefrontal cortex hypoactivity prevents compulsive cocaine seeking. Nature.

[57] Chen G, Goeddel DV (2002). TNF-R1 signaling: a beautiful pathway. Science.

[58] Chen Y, Zhao H, Ren X (2016). Estrogen and progestogen inhibit NF-kappaB in atherosclerotic tissues of ovariectomized ApoE (-/-) mice. Climacteric.

[59] Choi DW (1988). Glutamate neurotoxicity and diseases of the nervous system. Neuron.

[60] Chung IY, Benveniste EN (1990). Tumor necrosis factor-alpha production by astrocytes. Induction by lipopolysaccharide, IFN-gamma, and IL-1 beta. J Immunol.

[61] Conrad KL, Tseng KY, Uejima JL, Reimers JM, Heng LJ, Shaham Y, Marinelli M, Wolf ME (2008). Formation of accumbens GluR2-lacking AMPA receptors mediates incubation of cocaine craving. Nature.

[62] Copeland AL, Peltier MR, Geiselman PJ (2017). Severity of menopausal symptoms and nicotine dependence amongst postmenopausal women smokers. J Smok Cessat.

[63] Corradin SB, Mauel J, Donini SD, Quattrocchi E, Ricciardi-Castagnoli P (1993). Inducible nitric oxide synthase activity of cloned murine microglial cells. Glia.

[64] Crews FT, Qin L, Sheedy D, Vetreno RP, Zou J (2013). High mobility group box 1/Toll-like receptor danger signaling increases brain neuroimmune activation in alcohol dependence. Biol Psychiatry.

[65] Crews FT, Vetreno RP (2018). Stress and alcohol priming of brain Toll-Like receptor signaling in alcohol use disorder. Alcohol Alcohol.

[66] Crews FT, Walter TJ, Coleman LG, Vetreno RP (2017). Toll-like receptor signaling and stages of addiction. Psychopharmacology.

[67] Cross AK, Woodroofe MN (1999). Chemokine modulation of matrix metalloproteinase and TIMP production in adult rat brain microglia and a human microglial cell line in vitro. Glia.

[68] Crowley T, Cryan JF, Downer EJ, O'Leary OF (2016). Inhibiting neuroinflammation: The role and therapeutic potential of GABA in neuro-immune interactions. Brain Behav Immun.

[69] Csolle C, Ando RD, Kittel A, Goloncser F, Baranyi M, Soproni K, Zelena D, Haller J, Nemeth T, Mocsai A, Sperlagh B (2013). The absence of P2X7 receptors (P2rx7) on non-haematopoietic cells leads to selective alteration in mood-related behaviour with dysregulated gene expression and stress reactivity in mice. Int J Neuropsychopharmacol.

[70] Cui C, Shurtleff D, Harris RA (2014). Neuroimmune mechanisms of alcohol and drug addiction. Int Rev Neurobiol.

[71] D'Abrantes S, Gratton S, Reynolds P, Kriechbaumer V, McKenna J, Barnard S, Clarke DT, Botchway SW (2018). Super-resolution nanoscopy imaging applied to DNA double-strand breaks. Radiat Res.

[72] Davis BM, Salinas-Navarro M, Cordeiro MF, Moons L, De Groef L (2017). Characterizing microglia activation: a spatial statistics approach to maximize information extraction. Sci Rep.

[73] Dantzer R (2018). Neuroimmune interactions: from the brain to the immune system and vice versa. Physiol Rev.

[74] Dantzer R, Kelley KW (2007). Twenty years of research on cytokine-induced sickness behavior. Brain Behav Immun.

[75] De Biase LM, Schuebel KE, Fusfeld ZH, Jair K, Hawes IA, Cimbro R, Zhang HY, Liu QR, Shen H, Xi ZX, Goldman D, Bonci A (2017). Local cues establish and maintain region-specific phenotypes of basal ganglia microglia. Neuron.

[76] Del Giudice M. Invisible designers: brain evolution through the lens of parasite manipulation. Q Rev Biol. 2020;95.

[77] Deng L, Wang C, Spencer E, Yang L, Braun A, You J, Slaughter C, Pickart C, Chen ZJ (2000). Activation of the IkappaB kinase complex by TRAF6 requires a dimeric ubiquitin-conjugating enzyme complex and a unique polyubiquitin chain. Cell.

[78] Dias C, Dietz D, Mazei-Robison M, Sun H, Damez-Werno D, Ferguson D, Wilkinson M, Magida J, Gao V, Neve R, Nestler EJ (2015). Dishevelled-2 regulates cocaine-induced structural plasticity and Rac1 activity in the nucleus accumbens. Neurosci Lett.

[79] Dietz DM, Sun H, Lobo MK, Cahill ME, Chadwick B, Gao V, Koo JW, Mazei-Robison MS, Dias C, Maze I, Damez-Werno D, Dietz KC, Scobie KN, Ferguson D, Christoffel D, Ohnishi Y, Hodes GE, Zheng Y, Neve RL, Hahn KM, Russo SJ, Nestler EJ (2012). Rac1 is essential in cocaine-induced structural plasticity of nucleus accumbens neurons. Nat Neurosci.

[80] Ding Y, Shi W, Xie G, Yu A, Wang Q, Zhang Z (2015). CX3CR1 Mediates Nicotine Withdrawal-Induced Hyperalgesia via Microglial P38 MAPK Signaling. Neurochem Res.

[81] Doble A (1999). The role of excitotoxicity in neurodegenerative disease: implications for therapy. Pharmacol Ther.

[82] Donnelly-Roberts DL, Jarvis MF (2007). Discovery of P2X7 receptor-selective antagonists offers new insights into P2X7 receptor function and indicates a role in chronic pain states. Br J Pharmacol.

[83] Dorfman MD, Krull JE, Douglass JD, Fasnacht R, Lara-Lince F, Meek TH, Shi X, Damian V, Nguyen HT, Matsen ME, Morton GJ, Thaler JP (2017). Sex differences in microglial CX3CR1 signalling determine obesity susceptibility in mice. Nat Commun.

[84] Doyle HH, Eidson LN, Sinkiewicz DM, Murphy AZ (2017). Sex differences in microglia activity within the periaqueductal gray of the rat: a potential mechanism driving the dimorphic effects of morphine. J Neurosci.

[85] Duivis HE, Vogelzangs N, Kupper N, de Jonge P, Penninx BW (2013). Differential association of somatic and cognitive symptoms of depression and anxiety with inflammation: findings from the Netherlands Study of Depression and Anxiety (NESDA). Psychoneuroendocrinology.

[86] Dzierba AL, Jelic S (2009). Chronic obstructive pulmonary disease in the elderly: an update on pharmacological management. Drugs Aging.

[87] Eidson LN, Inoue K, Young LJ, Tansey MG, Murphy AZ (2017). Toll-like receptor 4 mediates morphine-induced neuroinflammation and tolerance via soluble tumor necrosis factor signaling. Neuropsychopharmacology.

[88] Everitt BJ, Robbins TW (2005). Neural systems of reinforcement for drug addiction: from actions to habits to compulsion. Nat Neurosci.

[89] Eyo UB, Wu LJ (2013). Bidirectional microglia-neuron communication in the healthy brain. Neural Plast.

[90] Fazio F, Ulivieri M, Volpi C, Gargaro M, Fallarino F (2018). Targeting metabotropic glutamate receptors for the treatment of neuroinflammation. Curr Opin Pharmacol.

[91] Fernandes NC, Sriram U, Gofman L, Cenna JM, Ramirez SH, Potula R (2016). Methamphetamine alters microglial immune function through P2X7R signaling. J Neuroinflammation.

[92] Fernandes S, Salta S, Bravo J, Silva AP, Summavielle T (2016). Acetyl-L-carnitine prevents methamphetamine-induced structural damage on endothelial cells via ILK-related MMP-9 activity. Mol Neurobiol.

[93] Fernandez-Arjona MDM, Grondona JM, Fernandez-Llebrez P, Lopez-Avalos MD (2019). Microglial morphometric parameters correlate with the expression level of IL-1beta, and allow identifying different activated morphotypes. Front Cell Neurosci.

[94] Fernandez-Arjona MDM, Grondona JM, Granados-Duran P, Fernandez-Llebrez P, Lopez-Avalos MD (2017). Microglia morphological categorization in a rat model of neuroinflammation by hierarchical cluster and principal components analysis. Front Cell Neurosci.

[95] Franklin TR, Ehrman R, Lynch KG, Harper D, Sciortino N, O'Brien CP, Childress AR (2008). Menstrual cycle phase at quit date predicts smoking status in an NRT treatment trial: a retrospective analysis. J Women's Health (Larchmt).

[96] Gahring LC, Carlson NG, Kulmar RA, Rogers SW (1996). Neuronal expression of tumor necrosis factor alpha in the murine brain. Neuroimmunomodulation.

[97] Gallaher ZR, Ryu V, Herzog T, Ritter RC, Czaja K (2012). Changes in microglial activation within the hindbrain, nodose ganglia, and the spinal cord following subdiaphragmatic vagotomy. Neurosci Lett.

[98] Gao Z, Zhu Q, Zhang Y, Zhao Y, Cai L, Shields CB (2013). Reciprocal modulation between microglia and astrocyte in reactive gliosis following the CNS injury. Mol Neurobiol.

[99] Gass JT, Sinclair CM, Cleva RM, Widholm JJ, Olive MF (2011). Alcohol-seeking behavior is associated with increased glutamate transmission in basolateral amygdala and nucleus accumbens as measured by glutamate-oxidase-coated biosensors. Addict Biol.

[100] Gavrilov N, Golyagina I, Brazhe A, Scimemi A, Turlapov V, Semyanov A (2018). Astrocytic coverage of dendritic spines, dendritic shafts, and axonal boutons in hippocampal neuropil. Front Cell Neurosci.

[101] Gegelashvili G, Schousboe A (1997). High affinity glutamate transporters: regulation of expression and activity. Mol Pharmacol.

[102] Gentile TA, Simmons SJ, Tallarida CS, Su S, Rom S, Watson MN, Reitz AB, Potula R, Rawls SM (2019). Synthetic cathinone MDPV enhances reward function through purinergic P2X7 receptor-dependent pathway and increases P2X7 gene expression in nucleus accumbens. Drug Alcohol Depend.

[103] Gessi S, Borea PA, Bencivenni S, Fazzi D, Varani K, Merighi S (2016). The activation of mu-opioid receptor potentiates LPS-induced NF-kB promoting an inflammatory phenotype in microglia. FEBS Lett.

[104] Gill SS, Pulido OM (2001). Glutamate receptors in peripheral tissues: current knowledge, future research, and implications for toxicology. Toxicol Pathol.

[105] Gilmore TD (2006). Introduction to NF-kappaB: players, pathways, perspectives. Oncogene.

[106] Ginhoux F, Greter M, Leboeuf M, Nandi S, See P, Gokhan S, Mehler MF, Conway SJ, Ng LG, Stanley ER, Samokhvalov IM, Merad M. Fate mapping analysis reveals that adult microglia derive from primitive macrophages. Science 2010;330(6005):841–5. 10.1126/science.1194637 PMC3719181 20966214

[107] Gipson CD (2016). Treating addiction: unraveling the relationship between N-acetylcysteine, glial glutamate transport, and behavior. Biol Psychiatry.

[108] Gipson CD, Bimonte-Nelson HA. Interactions between reproductive transitions during aging and addiction: promoting translational crosstalk between different fields of research. Behav Pharmacol. 2020. 10.1097/FBP.0000000000000591 PMC7965232 32960852

[109] Gipson CD, Kupchik YM, Kalivas PW. Rapid, transient synaptic plasticity in addiction. Neuropharmacology. 2014;76(Pt B):276–86. 10.1016/j.neuropharm.2013.04.032 PMC3762905 23639436

[110] Gipson CD, Reissner KJ, Kupchik YM, Smith AC, Stankeviciute N, Hensley-Simon ME, Kalivas PW (2013). Reinstatement of nicotine seeking is mediated by glutamatergic plasticity. Proc Natl Acad Sci U S A.

[111] Gladkevich A, Korf J, Hakobyan VP, Melkonyan KV (2006). The peripheral GABAergic system as a target in endocrine disorders. Auton Neurosci.

[112] Gofman L, Cenna JM, Potula R (2014). P2X4 receptor regulates alcohol-induced responses in microglia. J NeuroImmune Pharmacol.

[113] Goldstein RZ, Volkow ND (2002). Drug addiction and its underlying neurobiological basis: neuroimaging evidence for the involvement of the frontal cortex. Am J Psychiatry.

[114] Gourley SL, Olevska A, Warren MS, Taylor JR, Koleske AJ (2012). Arg kinase regulates prefrontal dendritic spine refinement and cocaine-induced plasticity. J Neurosci.

[115] Graeber MB (2010). Changing face of microglia. Science.

[116] Gray KM, Watson NL, Carpenter MJ, Larowe SD (2010). N-acetylcysteine (NAC) in young marijuana users: an open-label pilot study. Am J Addict.

[117] Griffin WC, Ramachandra VS, Knackstedt LA, Becker HC (2015). Repeated cycles of chronic intermittent ethanol exposure increases basal glutamate in the nucleus accumbens of mice without affecting glutamate transport. Front Pharmacol.

[118] Gubert C, Fries GR, Pfaffenseller B, Ferrari P, Coutinho-Silva R, Morrone FB, Kapczinski F, Battastini AMO (2016). Role of P2X7 Receptor in an animal model of mania induced by D-amphetamine. Mol Neurobiol.

[119] Gundersen V, Storm-Mathisen J, Bergersen LH (2015). Neuroglial transmission. Physiol Rev.

[120] Guyon A (2014). CXCL12 chemokine and its receptors as major players in the interactions between immune and nervous systems. Front Cell Neurosci.

[121] Hagino Y, Kariura Y, Manago Y, Amano T, Wang B, Sekiguchi M, Nishikawa K, Aoki S, Wada K, Noda M (2004). Heterogeneity and potentiation of AMPA type of glutamate receptors in rat cultured microglia. Glia.

[122] Hamilton NB, Attwell D (2010). Do astrocytes really exocytose neurotransmitters?. Nat Rev Neurosci.

[123] Hanisch UK, Kettenmann H (2007). Microglia: active sensor and versatile effector cells in the normal and pathologic brain. Nat Neurosci.

[124] Harkonen PL, Vaananen HK (2006). Monocyte-macrophage system as a target for estrogen and selective estrogen receptor modulators. Ann N Y Acad Sci.

[125] Haseleu J, Anlauf E, Blaess S, Endl E, Derouiche A (2013). Studying subcellular detail in fixed astrocytes: dissociation of morphologically intact glial cells (DIMIGs). Front Cell Neurosci.

[126] Haugeto O, Ullensvang K, Levy LM, Chaudhry FA, Honore T, Nielsen M, Lehre KP, Danbolt NC (1996). Brain glutamate transporter proteins form homomultimers. J Biol Chem.

[127] He Y, Taylor N, Fourgeaud L, Bhattacharya A (2017). The role of microglial P2X7: modulation of cell death and cytokine release. J Neuroinflammation.

[128] Heiman A, Pallottie A, Heary RF, Elkabes S (2014). Toll-like receptors in central nervous system injury and disease: a focus on the spinal cord. Brain Behav Immun.

[129] Heindl S, Gesierich B, Benakis C, Llovera G, Duering M, Liesz A (2018). Automated morphological analysis of microglia after stroke. Front Cell Neurosci.

[130] Heller JP, Rusakov DA (2015). Morphological plasticity of astroglia: understanding synaptic microenvironment. Glia.

[131] Hinoi E, Ogita K, Takeuchi Y, Ohashi H, Maruyama T, Yoneda Y (2001). Characterization with [3H]quisqualate of group I metabotropic glutamate receptor subtype in rat central and peripheral excitable tissues. Neurochem Int.

[132] Hinwood M, Tynan RJ, Charnley JL, Beynon SB, Day TA, Walker FR (2013). Chronic stress induced remodeling of the prefrontal cortex: structural re-organization of microglia and the inhibitory effect of minocycline. Cereb Cortex.

[133] Hodes GE, Kana V, Menard C, Merad M, Russo SJ (2015). Neuroimmune mechanisms of depression. Nat Neurosci.

[134] Hohlfeld R, Kerschensteiner M, Meinl E (2007). Dual role of inflammation in CNS disease. Neurology.

[135] Horvath RJ, DeLeo JA (2009). Morphine enhances microglial migration through modulation of P2X4 receptor signaling. J Neurosci.

[136] Hsieh YC, Frink M, Hsieh CH, Choudhry MA, Schwacha MG, Bland KI, Chaudry IH (2007). Downregulation of migration inhibitory factor is critical for estrogen-mediated attenuation of lung tissue damage following trauma-hemorrhage. Am J Phys Lung Cell Mol Phys.

[137] Huang B, Bates M, Zhuang X (2009). Super-resolution fluorescence microscopy. Annu Rev Biochem.

[138] Huhn AS, Berry MS, Dunn KE (2018). Systematic review of sex-based differences in opioid-based effects. Int Rev Psychiatry.

[139] Huhn AS, Berry MS, Dunn KE (2019). Review: sex-based differences in treatment outcomes for persons with opioid use disorder. Am J Addict.

[140] Hutchinson MR, Northcutt AL, Hiranita T, Wang X, Lewis SS, Thomas J, van Steeg K, Kopajtic TA, Loram LC, Sfregola C, Galer E, Miles NE, Bland ST, Amat J, Rozeske RR, Maslanik T, Chapman TR, Strand KA, Fleshner M, Bachtell RK, Somogyi AA, Yin H, Katz JL, Rice KC, Maier SF, Watkins LR (2012). Opioid activation of toll-like receptor 4 contributes to drug reinforcement. J Neurosci.

[141] Hutchinson MR, Zhang Y, Shridhar M, Evans JH, Buchanan MM, Zhao TX, Slivka PF, Coats BD, Rezvani N, Wieseler J, Hughes TS, Landgraf KE, Chan S, Fong S, Phipps S, Falke JJ, Leinwand LA, Maier SF, Yin H, Rice KC, Watkins LR (2010). Evidence that opioids may have toll-like receptor 4 and MD-2 effects. Brain Behav Immun.

[142] Ito D, Tanaka K, Suzuki S, Dembo T, Fukuuchi Y (2001). Enhanced expression of Iba1, ionized calcium-binding adapter molecule 1, after transient focal cerebral ischemia in rat brain. Stroke.

[143] Izeddin I, Specht CG, Lelek M, Darzacq X, Triller A, Zimmer C, Dahan M (2011). Super-resolution dynamic imaging of dendritic spines using a low-affinity photoconvertible actin probe. PLoS One.

[144] Jackson JC, Walker RF, Brooks WH, Roszman TL (1988). Specific uptake of serotonin by murine macrophages. Life Sci.

[145] Janeway CA, Medzhitov R (2002). Innate immune recognition. Annu Rev Immunol.

[146] Jastrzebska J, Nowak E, Smaga I, Bystrowska B, Frankowska M, Bader M, Filip M, Fuxe K (2014). Adenosine (A)(2A) receptor modulation of nicotine-induced locomotor sensitization. Pharmacol Transgenic Approach Neuropharmacol.

[147] Jelinek HF, Milošević NT, Karperien A, Krstonošić B (2013). Box-counting and multifractal analysis in neuronal and glial classification. Adv Intell Syst Compu.

[148] John GR, Simpson JE, Woodroofe MN, Lee SC, Brosnan CF (2001). Extracellular nucleotides differentially regulate interleukin-1beta signaling in primary human astrocytes: implications for inflammatory gene expression. J Neurosci.

[149] Kaindl AM, Degos V, Peineau S, Gouadon E, Chhor V, Loron G, Le CT, Josserand J, Ali C, Vivien D, Collingridge GL, Lombet A, Issa L, Rene F, Loeffler JP, Kavelaars A, Verney C, Mantz J, Gressens P (2012). Activation of microglial N-methyl-d-aspartate receptors triggers inflammation and neuronal cell death in the developing and mature brain. Ann Neurol.

[150] Kalaitzidis D, Gilmore TD (2005). Transcription factor cross-talk: the estrogen receptor and NF-kappaB. Trends Endocrinol Metab.

[151] Kalivas PW (2009). The glutamate homeostasis hypothesis of addiction. Nat Rev Neurosci.

[152] Kalivas PW, Volkow N, Seamans J (2005). Unmanageable motivation in addiction: a pathology in prefrontal-accumbens glutamate transmission. Neuron.

[153] Kaltschmidt B, Kaltschmidt C (2015). NF-KappaB in long-term memory and structural plasticity in the adult mammalian brain. Front Mol Neurosci.

[154] Kaltschmidt B, Uherek M, Volk B, Baeuerle PA, Kaltschmidt C (1997). Transcription factor NF-kappaB is activated in primary neurons by amyloid beta peptides and in neurons surrounding early plaques from patients with Alzheimer disease. Proc Natl Acad Sci U S A.

[155] Kaltschmidt B, Widera D, Kaltschmidt C (2005). Signaling via NF-kappaB in the nervous system. Biochim Biophys Acta.

[156] Kaltschmidt C, Kaltschmidt B, Baeuerle PA (1995). Stimulation of ionotropic glutamate receptors activates transcription factor NF-kappa B in primary neurons. Proc Natl Acad Sci U S A.

[157] Kano S, Choi EY, Dohi E, Agarwal S, Chang DJ, Wilson AM, Lo BD, Rose IVL, Imai T, Sawa A (2019). Glutathione S-transferases promote proinflammatory astrocyte-microglia communication during brain inflammation. Sci Signal.

[158] Karperien A, Ahammer H, Jelinek HF (2013). Quantitating the subtleties of microglial morphology with fractal analysis. Front Cell Neurosci.

[159] Kashima DT, Grueter BA (2017). Toll-like receptor 4 deficiency alters nucleus accumbens synaptic physiology and drug reward behavior. PNAS.

[160] Kassis JA, Gorski J (1981). Estrogen receptor replenishment. Evidence for receptor recycling. J Biol Chem.

[161] Kavetsky L, Green KK, Boyle BR, Yousufzai FAK, Padron ZM, Melli SE, Kuhnel VL, Jackson HM, Blanco RE, Howell GR, Soto I (2019). Increased interactions and engulfment of dendrites by microglia precede Purkinje cell degeneration in a mouse model of Niemann Pick Type-C. Sci Rep.

[162] Kawai T, Akira S (2011). Toll-like receptors and their crosstalk with other innate receptors in infection and immunity. Immunity.

[163] Kettenmann H, Kirchhoff F, Verkhratsky A (2013). Microglia: new roles for the synaptic stripper. Neuron.

[164] Khakh BS, Sofroniew MV (2015). Diversity of astrocyte functions and phenotypes in neural circuits. Nat Neurosci.

[165] Kim J, Connelly KL, Unterwald EM, Rawls SM (2017). Chemokines and cocaine: CXCR4 receptor antagonist AMD3100 attenuates cocaine place preference and locomotor stimulation in rats. Brain Behav Immun.

[166] Kim J, Kwon YH, Kim CS, Tu TH, Kim BS, Joe Y (2018). The involvement of 4-1BB/4-1BBL signaling in glial cell-mediated hypothalamic inflammation in obesity. FEBS Open Bio.

[167] Kipnis J (2016). Multifaceted interactions between adaptive immunity and the central nervous system. Science.

[168] Knackstedt LA, Melendez RI, Kalivas PW (2010). Ceftriaxone restores glutamate homeostasis and prevents relapse to cocaine seeking. Biol Psychiatry.

[169] Kondo S, Kohsaka S, Okabe S (2011). Long-term changes of spine dynamics and microglia after transient peripheral immune response triggered by LPS in vivo. Mol Brain.

[170] Kongsui R, Beynon SB, Johnson SJ, Walker FR (2014). Quantitative assessment of microglial morphology and density reveals remarkable consistency in the distribution and morphology of cells within the healthy prefrontal cortex of the rat. J Neuroinflammation.

[171] Koo JW, Russo SJ, Ferguson D, Nestler EJ, Duman RS (2010). Nuclear factor-kappaB is a critical mediator of stress-impaired neurogenesis and depressive behavior. Proc Natl Acad Sci U S A.

[172] Koob GF, Nestler EJ (1997). The neurobiology of drug addiction. J Neuropsychiatr Clin Neurosci.

[173] Koss K, Churchward MA, Tsui C, Todd KG (2019). In vitro priming and hyper-activation of brain microglia: an assessment of phenotypes. Mol Neurobiol.

[174] Krugel U (2016). Purinergic receptors in psychiatric disorders. Neuropharmacology.

[175] Kruyer A, Scofield MD, Wood D, Reissner KJ, Kalivas PW (2019). Heroin cue-evoked astrocytic structural plasticity at nucleus accumbens synapses inhibits heroin seeking. Biol Psychiatry.

[176] Kunkler PE, Kraig RP (1997). Reactive astrocytosis from excitotoxic injury in hippocampal organ culture parallels that seen in vivo. J Cereb Blood Flow Metab.

[177] LaLumiere RT, Kalivas PW (2008). Glutamate release in the nucleus accumbens core is necessary for heroin seeking. J Neurosci.

[178] Larowe SD, Kalivas PW, Nicholas JS, Randall PK, Mardikian PN, Malcolm RJ (2013). A double-blind placebo-controlled trial of N-acetylcysteine in the treatment of cocaine dependence. Ame J Addict.

[179] Lau A, Tymianski M (2010). Glutamate receptors, neurotoxicity and neurodegeneration. Pflugers Arch.

[180] Lee E, Sidoryk-Wegrzynowicz M, Yin Z, Webb A, Son DS, Aschner M (2012). Transforming growth factor-alpha mediates estrogen-induced upregulation of glutamate transporter GLT-1 in rat primary astrocytes. Glia.

[181] Lee H, Lee S, Cho I, Lee SJ (2013). Toll-like receptors: sensor molecules for detecting damage to the nervous system. Curr Protein Pept Sci.

[182] Lee SJ, Zhou T, Choi C, Wang Z, Benveniste EN (2000). Differential regulation and function of Fas expression on glial cells. J Immunol.

[183] Leon-Ponte M, Ahern GP, O'Connell PJ (2007). Serotonin provides an accessory signal to enhance T-cell activation by signaling through the 5-HT7 receptor. Blood.

[184] Lewitus GM, Konefal SC, Greenhalgh AD, Pribiag H, Augereau K, Stellwagen D (2016). Microglial TNF-alpha suppresses cocaine-induced plasticity and behavioral sensitization. Neuron.

[185] Li Q, Barres BA (2018). Microglia and macrophages in brain homeostasis and disease. Nat Rev Immunol.

[186] Li TT, Zhu D, Mou T, Guo Z, Pu JL, Chen QS, Wei XF, Wu ZJ (2017). IL-37 induces autophagy in hepatocellular carcinoma cells by inhibiting the PI3K/AKT/mTOR pathway. Mol Immunol.

[187] Liao K, Guo M, Niu F, Yang L, Callen SE, Buch S (2016). Cocaine-mediated induction of microglial activation involves the ER stress-TLR2 axis. J Neuroinflammation.

[188] Liddie S, Anderson KL, Paz A, Itzhak Y (2012). The effect of phosphodiesterase inhibitors on the extinction of cocaine-induced conditioned place preference in mice. J Psychopharmacol.

[189] Liu ZG (2005). Molecular mechanism of TNF signaling and beyond. Cell Res.

[190] Loftis JM, Janowsky A (2014). Neuroimmune basis of methamphetamine toxicity. Int Rev Neurobiol.

[191] Longcope C, Kato T, Horton R (1969). Conversion of blood androgens to estrogens in normal adult men and women. J Clin Invest.

[192] Lopez-Redondo F, Nakajima K, Honda S, Kohsaka S (2000). Glutamate transporter GLT-1 is highly expressed in activated microglia following facial nerve axotomy. Brain Res Mol Brain Res.

[193] Lu B (2004). Acute and long-term synaptic modulation by neurotrophins. Prog Brain Res.

[194] Luscher C, Robbins TW, Everitt BJ (2020). The transition to compulsion in addiction. Nat Rev Neurosci.

[195] Luskin MB, Parnavelas JG, Barfield JA (1993). Neurons, astrocytes, and oligodendrocytes of the rat cerebral cortex originate from separate progenitor cells: an ultrastructural analysis of clonally related cells. J Neurosci.

[196] Lynch WJ, Arizzi MN, Carroll ME (2000). Effects of sex and the estrous cycle on regulation of intravenously self-administered cocaine in rats. Psychopharmacology.

[197] Machado R, Vargas HO, Baracat MM, Urbano MR, Verri WA Jr, Porcu M, Nunes SOV. N-acetylcysteine as an adjunctive treatment for smoking cessation: a randomized clinical trial. Braz J Psychiatry. 2020. 10.1590/1516-4446-2019-0753 PMC7524413 32725102

[198] Macht VA (2016). Neuro-immune interactions across development: a look at glutamate in the prefrontal cortex. Neurosci Biobehav Rev.

[199] Maduna T, Audouard E, Dembele D, Mouzaoui N, Reiss D, Massotte D, Gaveriaux-Ruff C (2018). Microglia express mu opioid receptor: insights from transcriptomics and fluorescent reporter mice. Front Psychiatry.

[200] Mastrangelo, F., Frydas, I., Ronconi, G., Kritas, S.K., Tettamanti, L., Caraffa, A., C, D.O., Younes, A., Gallenga, C.E., Conti, P., 2018. Low-grade chronic inflammation mediated by mast cells in fibromyalgia: role of IL-37. J Biol Regul Homeost Agents 32, 195-198. 29684996

[201] Matejuk A, Ransohoff RM (2020). Crosstalk between astrocytes and microglia: an overview. Front Immunol.

[202] McClure EA, Baker NL, Gipson CD, Carpenter MJ, Roper AP, Froeliger BE, Kalivas PW, Gray KM. An open-label pilot trial of N-acetylcysteine and varenicline in adult cigarette smokers. Am J Drug Alcohol Abuse. 2014:1–5. 10.3109/00952990.2014.933839 PMC4262740 25062287

[203] McCutcheon JE, Wang X, Tseng KY, Wolf ME, Marinelli M (2011). Calcium-permeable AMPA receptors are present in nucleus accumbens synapses after prolonged withdrawal from cocaine self-administration but not experimenter-administered cocaine. J Neurosci.

[204] McDonnell DP, Norris JD (2002). Connections and regulation of the human estrogen receptor. Science.

[205] McFarland K, Lapish CC, Kalivas PW (2003). Prefrontal glutamate release into the core of the nucleus accumbens mediates cocaine-induced reinstatement of drug-seeking behavior. J Neurosci.

[206] Medzhitov R, Preston-Hurlburt P, Janeway CA (1997). A human homologue of the Drosophila Toll protein signals activation of adaptive immunity. Nature.

[207] Meffert MK, Chang JM, Wiltgen BJ, Fanselow MS, Baltimore D (2003). NF-kappa B functions in synaptic signaling and behavior. Nat Neurosci.

[208] Meissner A, Visanji NP, Momen MA, Feng R, Francis BM, Bolz SS, Hazrati LN. Tumor necrosis factor-alpha underlies loss of cortical dendritic spine density in a mouse model of congestive heart failure. J Am Heart Assoc. 2015;4. 10.1161/JAHA.115.001920 PMC4599420 25948533

[209] Men Y, Yelick J, Jin S, Tian Y, Chiang MSR, Higashimori H, Brown E, Jarvis R, Yang Y (2019). Exosome reporter mice reveal the involvement of exosomes in mediating neuron to astroglia communication in the CNS. Nat Commun.

[210] Menard C, Pfau ML, Hodes GE, Russo SJ (2017). Immune and neuroendocrine mechanisms of stress vulnerability and resilience. Neuropsychopharmacology.

[211] Meng P, Chen ZG, Zhang TT, Liang ZZ, Zou XL, Yang HL, Li HT (2019). IL-37 alleviates house dust mite-induced chronic allergic asthma by targeting TSLP through the NF-kappaB and ERK1/2 signaling pathways. Immunol Cell Biol.

[212] Merlini M, Rafalski VA, Ma K, Kim K, Bushong EA, Rios Coronado PE, Yan Z, Mendiola AS, Sozmen E, Ryu JK, Haberl MG, Madany M, Sampson DN, Petersen MA, Bardehle S, Tognatta R, Dean T Jr, Meza Acevedo R, Cabriga B, Thomas R, Coughlin SR, Ellisman MH, Palop JJ, Akassoglou K. Microglial G i-dependent dynamics regulate brain network hyperexcitability. Nat Neurosci. 2020; Online ahead of print. 10.1038/s41593-020-00756-7 PMC8118167 33318667

[213] Meskini N, Nemoz G, Okyayuz-Baklouti I, Lagarde M, Prigent AF (1994). Phosphodiesterase inhibitory profile of some related xanthine derivatives pharmacologically active on the peripheral microcirculation. Biochem Pharmacol.

[214] Miller EC, Zhang L, Dummer BW, Cariveau DR, Loh H, Law PY, Liao D (2012). Differential modulation of drug-induced structural and functional plasticity of dendritic spines. Mol Pharmacol.

[215] Minter MR, Taylor JM, Crack PJ (2016). The contribution of neuroinflammation to amyloid toxicity in Alzheimer's disease. J Neurochem.

[216] Mishra D, Pena-Bravo JI, Leong KC, Lavin A, Reichel CM (2017). Methamphetamine self-administration modulates glutamate neurophysiology. Brain Struct Funct.

[217] Monasterio N, Vergara E, Morales T (2013). Hormonal influences on neuroimmune responses in the CNS of females. Front Integr Neurosci.

[218] Montagud-Romero S, Montesinos J, Pavon FJ, Blanco-Gandia MC, Ballestin R, Rodriguez de Fonseca F, Minarro J, Guerri C, Rodriguez-Arias M (2020). Social defeat-induced increase in the conditioned rewarding effects of cocaine: Role of CX3CL1. Prog Neuro-Psychopharmacol Biol Psychiatry.

[219] Montesinos J, Pascual M, Rodriguez-Arias M, Minarro J, Guerri C (2016). Involvement of TLR4 in the long-term epigenetic changes, rewarding and anxiety effects induced by intermittent ethanol treatment in adolescence. Brain Behav Immun.

[220] Morale MC, Serra PA, L'Episcopo F, Tirolo C, Caniglia S, Testa N, Gennuso F, Giaquinta G, Rocchitta G, Desole MS, Miele E, Marchetti B (2006). Estrogen, neuroinflammation and neuroprotection in Parkinson’s disease: glia dictates resistance versus vulnerability to neurodegeneration. Neuroscience.

[221] Moretti S, Bozza S, Oikonomou V, Renga G, Casagrande A, Iannitti RG, Puccetti M, Garlanda C, Kim S, Li S, van de Veerdonk FL, Dinarello CA, Romani L (2014). IL-37 inhibits inflammasome activation and disease severity in murine aspergillosis. PLoS Pathog.

[222] Morrison H, Young K, Qureshi M, Rowe RK, Lifshitz J (2017). Quantitative microglia analyses reveal diverse morphologic responses in the rat cortex after diffuse brain injury. Sci Rep.

[223] Morrison HW, Filosa JA (2013). A quantitative spatiotemporal analysis of microglia morphology during ischemic stroke and reperfusion. J Neuroinflammation.

[224] Moussawi K, Pacchioni A, Moran M, Olive MF, Gass JT, Lavin A, Kalivas PW (2009). N-Acetylcysteine reverses cocaine-induced metaplasticity. Nat Neurosci.

[225] Mueck AO, Seeger H (2005). Smoking, estradiol metabolism and hormone replacement therapy. Curr Med Chem Cardiovasc Hematol Agents.

[226] Muir RL (2009). Peripheral arterial disease: Pathophysiology, risk factors, diagnosis, treatment, and prevention. J Vasc Nurs.

[227] Nakajima K, Tohyama Y, Kohsaka S, Kurihara T (2001). Ability of rat microglia to uptake extracellular glutamate. Neurosci Lett.

[228] Namba MD, Kupchik YM, Spencer SM, Garcia-Keller C, Goenaga JG, Powell GL, Vicino IA, Hogue IB, Gipson CD. Accumbens neuroimmune signaling and dysregulation of astrocytic glutamate transport underlie conditioned nicotine-seeking behavior. Addict Biol. 2019:e12797. 10.1111/adb.12797 PMC7323912 31330570

[229] Narita M, Suzuki M, Kuzumaki N, Miyatake M, Suzuki T (2008). Implication of activated astrocytes in the development of drug dependence: differences between methamphetamine and morphine. Ann N Y Acad Sci.

[230] Nedergaard M, Takano T, Hansen AJ (2002). Beyond the role of glutamate as a neurotransmitter. Nat Rev Neurosci.

[231] Nedergaard M, Ransom B, Goldman SA (2003). New roles for astrocytes: redefining the functional architecture of the brain. Trends Neurosci.

[232] Nimmerjahn A, Kirchhoff F, Helmchen F (2005). Resting microglial cells are highly dynamic surveillants of brain parenchyma in vivo. Science.

[233] Nishida H, Okabe S (2007). Direct astrocytic contacts regulate local maturation of dendritic spines. J Neurosci.

[234] Noda M, Nakanishi H, Nabekura J, Akaike N (2000). AMPA-kainate subtypes of glutamate receptor in rat cerebral microglia. J Neurosci.

[235] Northcutt AL, Hutchinson MR, Wang X, Baratta MV, Hiranita T, Cochran TA, Pomrenze MB, Galer EL, Kopajtic TA, Li CM, Amat J, Larson G, Cooper DC, Huang Y, O'Neill CE, Yin H, Zahniser NR, Katz JL, Rice KC, Maier SF, Bachtell RK, Watkins LR (2015). DAT isn’t all that: cocaine reward and reinforcement require Toll-like receptor 4 signaling. Mol Psychiatry.

[236] O'Neill CE, LeTendre ML, Bachtell RK (2012). Adenosine A2A receptors in the nucleus accumbens bi-directionally alter cocaine seeking in rats. Neuropsychopharmacology.

[237] O'Neill LA, Kaltschmidt C (1997). NF-kappa B: a crucial transcription factor for glial and neuronal cell function. Trends Neurosci.

[238] Ogoshi F, Yin HZ, Kuppumbatti Y, Song B, Amindari S, Weiss JH (2005). Tumor necrosis-factor-alpha (TNF-alpha) induces rapid insertion of Ca2 + -permeable alpha-amino-3-hydroxyl-5-methyl-4-isoxazole-propionate (AMPA)/kainate (Ca-A/K) channels in a subset of hippocampal pyramidal neurons. Exp Neurol.

[239] Oliver CF, Simmons SJ, Nayak SU, Smith GR, Reitz AB, Rawls SM (2018). Chemokines and 'bath salts': CXCR4 receptor antagonist reduces rewarding and locomotor-stimulant effects of the designer cathinone MDPV in rats. Drug Alcohol Depend.

[240] Olmos G, Llado J (2014). Tumor necrosis factor alpha: a link between neuroinflammation and excitotoxicity. Mediat Inflamm.

[241] Ouali Alami N, Schurr C, Olde Heuvel F, Tang L, Li Q, Tasdogan A, Kimbara A, Nettekoven M, Ottaviani G, Raposo C, Rover S, Rogers-Evans M, Rothenhausler B, Ullmer C, Fingerle J, Grether U, Knuesel I, Boeckers TM, Ludolph A, Wirth T, Roselli F, Baumann B. NF-kappaB activation in astrocytes drives a stage-specific beneficial neuroimmunological response in ALS. EMBO J. 2018;37. 10.15252/embj.201798697 PMC6092622 29875132

[242] Pascual O, Ben Achour S, Rostaing P, Triller A, Bessis A (2012). Microglia activation triggers astrocyte-mediated modulation of excitatory neurotransmission. Proc Natl Acad Sci U S A.

[243] Pawlak J, Brito V, Kuppers E, Beyer C (2005). Regulation of glutamate transporter GLAST and GLT-1 expression in astrocytes by estrogen. Brain Res Mol Brain Res.

[244] Pedras-Vasconcelos J, Puig M, Verthelyi D (2009). TLRs as therapeutic targets in CNS inflammation and infection. Front Biosci (Elite Ed).

[245] Pena-Ortega F (2017). Pharmacological tools to activate microglia and their possible use to study neural network patho-physiology. Curr Neuropharmacol.

[246] Peng H, Geil Nickell CR, Chen KY, McClain JA, Nixon K (2017). Increased expression of M1 and M2 phenotypic markers in isolated microglia after four-day binge alcohol exposure in male rats. Alcohol.

[247] Peng H, Nixon K. Microglia phenotype is not as simple as M1- or M2-like after alcohol dependence in adolescent rats. Alcohol Clin Exp Res. 2020. 10.1111/acer.14504 PMC8296648 33164228

[248] Periyasamy P, Liao K, Kook YH, Niu F, Callen SE, Guo ML, Buch S (2018). Cocaine-mediated downregulation of miR-124 activates microglia by targeting KLF4 and TLR4 signaling. Mol Neurobiol.

[249] Perkins KA, Donny E, Caggiula AR (1999). Sex differences in nicotine effects and self-administration: review of human and animal evidence. Nicotine Tob Res.

[250] Piper ME, Federman EB, McCarthy DE, Bolt DM, Smith SS, Fiore MC, Baker TB (2007). Efficacy of bupropion alone and in combination with nicotine gum. Nicotine Tob Res.

[251] Ragozzino D, Renzi M, Giovannelli A, Eusebi F (2002). Stimulation of chemokine CXC receptor 4 induces synaptic depression of evoked parallel fibers inputs onto Purkinje neurons in mouse cerebellum. J Neuroimmunol.

[252] Ransohoff RM, Liu L, Cardona AE (2007). Chemokines and chemokine receptors: multipurpose players in neuroinflammation. Int Rev Neurobiol.

[253] Rathinam VA, Vanaja SK, Fitzgerald KA (2012). Regulation of inflammasome signaling. Nat Immunol.

[254] Reissner KJ, Gipson CD, Tran PK, Knackstedt LA, Scofield MD, Kalivas PW (2015). Glutamate transporter GLT-1 mediates N-acetylcysteine inhibition of cocaine reinstatement. Addict Biol.

[255] Roberts-Wolfe DJ, Kalivas PW (2015). Glutamate transporter GLT-1 as a therapeutic target for substance use disorders. CNS Neurol Disord Drug Targets.

[256] Rubio-Araiz A, Porcu F, Perez-Hernandez M, Garcia-Gutierrez MS, Aracil-Fernandez MA, Gutierrez-Lopez MD, Guerri C, Manzanares J, O'Shea E, Colado MI (2017). Disruption of blood-brain barrier integrity in postmortem alcoholic brain: preclinical evidence of TLR4 involvement from a binge-like drinking model. Addict Biol.

[257] Ruby CL, Adams CA, Knight EJ, Nam HW, Choi DS (2010). An essential role for adenosine signaling in alcohol abuse. Curr Drug Abuse Rev.

[258] Russo SJ, Mazei-Robison MS, Ables JL, Nestler EJ (2009). Neurotrophic factors and structural plasticity in addiction. Neuropharmacology.

[259] Russo SJ, Wilkinson MB, Mazei-Robison MS, Dietz DM, Maze I, Krishnan V, Renthal W, Graham A, Birnbaum SG, Green TA, Robison B, Lesselyong A, Perrotti LI, Bolanos CA, Kumar A, Clark MS, Neumaier JF, Neve RL, Bhakar AL, Barker PA, Nestler EJ (2009). Nuclear factor kappa B signaling regulates neuronal morphology and cocaine reward. J Neurosci.

[260] Sanchez R, Nguyen D, Rocha W, White JH, Mader S (2002). Diversity in the mechanisms of gene regulation by estrogen receptors. Bioessays.

[261] Santello M, Bezzi P, Volterra A (2011). TNFalpha controls glutamatergic gliotransmission in the hippocampal dentate gyrus. Neuron.

[262] Sari Y, Smith KD, Ali PK, Rebec GV (2009). Upregulation of GLT1 attenuates cue-induced reinstatement of cocaine-seeking behavior in rats. J Neurosci.

[263] Savage JC, Picard K, Gonzalez-Ibanez F, Tremblay ME (2018). A brief history of microglial ultrastructure: distinctive features, phenotypes, and functions discovered over the past 60 years by electron microscopy. Front Immunol.

[264] Sawada K, Kawakami R, Shigemoto R, Nemoto T (2018). Super-resolution structural analysis of dendritic spines using three-dimensional structured illumination microscopy in cleared mouse brain slices. Eur J Neurosci.

[265] Schafer DP, Lehrman EK, Kautzman AG, Koyama R, Mardinly AR, Yamasaki R (2012). Microglia sculpt postnatal neural circuits in an activity and complement-dependent manner. Neuron.

[266] Schmidt M, Hartung R, Capellino S, Cutolo M, Pfeifer-Leeg A, Straub RH. Estradiol Conversion to, and Tumor Necrosis Factor Inhibition by, Estrogen Metabolites in Synovial Cells of Patients With Rheumatoid Arthritis and Patients With Osteoarthritis. Arthritis Rheum. 2009;60(10):2913–22. 10.1002/art.24859 19790073

[267] Schroeder JA, Ruta JD, Gordon JS, Rodrigues AS, Foote CC (2012). The phosphodiesterase inhibitor isobutylmethylxanthine attenuates behavioral sensitization to cocaine. Behav Pharmacol.

[268] Scofield MD (2018). Exploring the role of astroglial glutamate release and association with synapses in neuronal function and behavior. Biol Psychiatry.

[269] Scofield MD, Heinsbroek JA, Gipson CD, Kupchik YM, Spencer S, Smith AC, Roberts-Wolfe D, Kalivas PW (2016). The nucleus accumbens: mechanisms of addiction across drug classes reflect the importance of glutamate homeostasis. Pharmacol Rev.

[270] Scofield MD, Kalivas PW (2014). Astrocytic dysfunction and addiction: consequences of impaired glutamate homeostasis. Neuroscientist.

[271] Scofield MD, Li H, Siemsen BM, Healey KL, Tran PK, Woronoff N, Boger HA, Kalivas PW, Reissner KJ (2016). Cocaine self-administration and extinction leads to reduced glial fibrillary acidic protein expression and morphometric features of astrocytes in the nucleus accumbens core. Biol Psychiatry.

[272] Shen HW, Scofield MD, Boger H, Hensley M, Kalivas PW (2014). Synaptic glutamate spillover due to impaired glutamate uptake mediates heroin relapse. J Neurosci.

[273] Shigetomi E, Kracun S, Khakh BS (2010). Monitoring astrocyte calcium microdomains with improved membrane targeted GCaMP reporters. Neuron Glia Biol.

[274] Shigetomi E, Kracun S, Sofroniew MV, Khakh BS (2010). A genetically targeted optical sensor to monitor calcium signals in astrocyte processes. Nat Neurosci.

[275] Siemsen BM, Giannotti G, McFaddin JA, Scofield MD, McGinty JF. Biphasic effect of abstinence duration following cocaine self-administration on spine morphology and plasticity-related proteins in prelimbic cortical neurons projecting to the nucleus accumbens core. Brain Struct Funct. 2018. 10.1007/s00429-018-1805-z PMC6438710 30498893

[276] Siemsen BM, McFaddin JA, Haigh K, Brock AG, Nan Leath M, Hooker KN, McGonegal LK, Scofield MD. Amperometric measurements of cocaine cue and novel context-evoked glutamate and nitric oxide release in the nucleus accumbens core. J Neurochem. 2020:e14952. 10.1111/jnc.14952 PMC7593647 31901130

[277] Siemsen BM, Reichel CM, Leong KC, Garcia-Keller C, Gipson CD, Spencer S, McFaddin JA, Hooker KN, Kalivas PW, Scofield MD (2019). Effects of methamphetamine self-administration and extinction on astrocyte structure and function in the nucleus accumbens core. Neuroscience.

[278] Sitcheran R, Gupta P, Fisher PB, Baldwin AS (2005). Positive and negative regulation of EAAT2 by NF-kappaB: a role for N-myc in TNFalpha-controlled repression. EMBO J.

[279] Skrzydelski D, Guyon A, Dauge V, Rovere C, Apartis E, Kitabgi P, Nahon JL, Rostene W, Parsadaniantz SM (2007). The chemokine stromal cell-derived factor-1/CXCL12 activates the nigrostriatal dopamine system. J Neurochem.

[280] Smaga I, Fierro D, Mesa J, Filip M, Knackstedt LA (2020). Molecular changes evoked by the beta-lactam antibiotic ceftriaxone across rodent models of substance use disorder and neurological disease. Neurosci Biobehav Rev.

[281] Smith AC, Kupchik YM, Scofield MD, Gipson CD, Wiggins A, Thomas CA, Kalivas PW (2014). Synaptic plasticity mediating cocaine relapse requires matrix metalloproteinases. Nat Neurosci.

[282] Smith AC, Scofield MD, Kalivas PW (2015). The tetrapartite synapse: extracellular matrix remodeling contributes to corticoaccumbens plasticity underlying drug addiction. Brain Res.

[283] Smith ACW, Scofield MD, Heinsbroek JA, Gipson CD, Neuhofer D, Roberts-Wolfe DJ, Spencer S, Garcia-Keller C, Stankeviciute NM, Smith RJ, Allen NP, Lorang MR, Griffin WC, Boger HA, Kalivas PW (2017). Accumbens nNOS interneurons regulate cocaine relapse. J Neurosci.

[284] Sofroniew MV, Vinters HV (2010). Astrocytes: biology and pathology. Acta Neuropathol.

[285] Sofuoglu M, Babb DA, Hatsukami DK (2001). Progesterone treatment during the early follicular phase of the menstrual cycle: effects on smoking behavior in women. Pharmacol Biochem Behav.

[286] Sofuoglu M, Babb DA, Hatsukami DK (2002). Effects of progesterone treatment on smoked cocaine response in women. Pharmacol Biochem Behav.

[287] Sofuoglu M, Mitchell E, Kosten TR (2004). Effects of progesterone treatment on cocaine responses in male and female cocaine users. Pharmacol Biochem Behav.

[288] Sofuoglu M, Mitchell E, Mooney M (2009). Progesterone effects on subjective and physiological responses to intravenous nicotine in male and female smokers. Hum Psychopharmacol.

[289] Sparacio SM, Zhang Y, Vilcek J, Benveniste EN (1992). Cytokine regulation of interleukin-6 gene expression in astrocytes involves activation of an NF-kappa B-like nuclear protein. J Neuroimmunol.

[290] Spiga S, Talani G, Mulas G, Licheri V, Fois GR, Muggironi G, Masala N, Cannizzaro C, Biggio G, Sanna E, Diana M (2014). Hampered long-term depression and thin spine loss in the nucleus accumbens of ethanol-dependent rats. Proc Natl Acad Sci U S A.

[291] Stefanik MT, Moussawi K, Kupchik YM, Smith KC, Miller RL, Huff ML, Deisseroth K, Kalivas PW, LaLumiere RT (2013). Optogenetic inhibition of cocaine seeking in rats. Addict Biology.

[292] Stein B, Yang MX (1995). Repression of the interleukin-6 promoter by estrogen receptor is mediated by NF-kappa B and C/EBP beta. Mol Cell Biol.

[293] Stellwagen D, Beattie EC, Seo JY, Malenka RC (2005). Differential regulation of AMPA receptor and GABA receptor trafficking by tumor necrosis factor-alpha. J Neurosci.

[294] Stellwagen D, Malenka RC (2006). Synaptic scaling mediated by glial TNF-alpha. Nature.

[295] Stumm R, Kolodziej A, Schulz S, Kohtz JD, Hollt V (2007). Patterns of SDF-1alpha and SDF-1gamma mRNAs, migration pathways, and phenotypes of CXCR4-expressing neurons in the developing rat telencephalon. J Comp Neurol.

[296] Stuckey DJ, Anthony DC, Lowe JP, Miller J, Palm WM, Styles P, Perry VH, Blamire AM, Sibson NR (2005). Detection of the inhibitory neurotransmitter GABA in macrophages by magnetic resonance spectroscopy. J Leukoc Biol.

[297] Su ZZ, Leszczyniecka M, Kang DC, Sarkar D, Chao W, Volsky DJ, Fisher PB (2003). Insights into glutamate transport regulation in human astrocytes: cloning of the promoter for excitatory amino acid transporter 2 (EAAT2). Proc Natl Acad Sci U S A.

[298] Sun SH (2010). Roles of P2X7 receptor in glial and neuroblastoma cells: the therapeutic potential of P2X7 receptor antagonists. Mol Neurobiol.

[299] Sun X, Wolf ME (2009). Nucleus accumbens neurons exhibit synaptic scaling that is occluded by repeated dopamine pre-exposure. Eur J Neurosci.

[300] Suzumura A (2013). Neuron-microglia interaction in neuroinflammation. Curr Protein Pept Sci.

[301] Swanson RA, Liu J, Miller JW, Rothstein JD, Farrell K, Stein BA, Longuemare MC (1997). Neuronal regulation of glutamate transporter subtype expression in astrocytes. J Neurosci.

[302] Sweitzer SM, Schubert P, DeLeo JA (2001). Propentofylline, a glial modulating agent, exhibits antiallodynic properties in a rat model of neuropathic pain. J Pharmacol Exp Ther.

[303] Tannenbaum C, Schwarz JM, Clayton JA, de Vries GJ, Sullivan C (2016). Evaluating sex as a biological variable in preclinical research: the devil in the details. Biol Sex Differ.

[304] Tansavatdi K, McClain B, Herrington DM (2004). The effects of smoking on estradiol metabolism. Minerva Ginecol.

[305] Tawfik VL, Regan MR, Haenggeli C, Lacroix-Fralish ML, Nutile-McMenemy N, Perez N, Rothstein JD, DeLeo JA (2008). Propentofylline-induced astrocyte modulation leads to alterations in glial glutamate promoter activation following spinal nerve transection. Neuroscience.

[306] Taylor DL, Diemel LT, Pocock JM (2003). Activation of microglial group III metabotropic glutamate receptors protects neurons against microglial neurotoxicity. J Neurosci.

[307] Taylor DL, Jones F, Kubota ES, Pocock JM (2005). Stimulation of microglial metabotropic glutamate receptor mGlu2 triggers tumor necrosis factor alpha-induced neurotoxicity in concert with microglial-derived Fas ligand. J Neurosci.

[308] Testen A, Ali M, Sexton HG, Hodges S, Dubester K, Reissner KJ, Swartzwelder HS, Risher ML (2019). Region-specific differences in morphometric features and synaptic colocalization of astrocytes during development. Neuroscience.

[309] Testen A, Kim R, Reissner KJ (2020). High-resolution three-dimensional imaging of individual astrocytes using confocal microscopy. Curr Protoc Neurosci.

[310] Testen A, Sepulveda-Orengo MT, Gaines CH, Reissner KJ (2018). Region-specific reductions in morphometric properties and synaptic colocalization of astrocytes following cocaine self-administration and extinction. Front Cell Neurosci.

[311] Thoenen H (1995). Neurotrophins and neuronal plasticity. Science.

[312] Tian J, Lu Y, Zhang H, Chau CH, Dang HN, Kaufman DL (2004). Gamma-aminobutyric acid inhibits T cell autoimmunity and the development of inflammatory responses in a mouse type 1 diabetes model. J Immunol.

[313] Tilleux S, Hermans E (2007). Neuroinflammation and regulation of glial glutamate uptake in neurological disorders. J Neurosci Res.

[314] Torres-Platas SG, Comeau S, Rachalski A, Bo GD, Cruceanu C, Turecki G, Giros B, Mechawar N (2014). Morphometric characterization of microglial phenotypes in human cerebral cortex. J Neuroinflammation.

[315] Tozzi A, de Iure A, Marsili V, Romano R, Tantucci M, Di Filippo M, Costa C, Napolitano F, Mercuri NB, Borsini F, Giampa C, Fusco FR, Picconi B, Usiello A, Calabresi P (2012). A2A adenosine receptor antagonism enhances synaptic and motor effects of cocaine via CB1 cannabinoid receptor activation. PLoS One.

[316] Trecki J, Brailoiu GC, Unterwald EM (2010). Localization of CXCR4 in the forebrain of the adult rat. Brain Res.

[317] Trecki J, Unterwald EM (2009). Modulation of cocaine-induced activity by intracerebral administration of CXCL12. Neuroscience.

[318] Tucker P, Ruwe WD, Masters B, Parker DE, Hossain A, Trautman RP, Wyatt DB (2004). Neuroimmune and cortisol changes in selective serotonin reuptake inhibitor and placebo treatment of chronic posttraumatic stress disorder. Biol Psychiatry.

[319] Tzagarakis-Foster C, Geleziunas R, Lomri A, An J, Leitman DC (2002). Estradiol represses human T-cell leukemia virus type 1 Tax activation of tumor necrosis factor-alpha gene transcription. J Biol Chem.

[320] Ucha, M., Roura-Martinez, D., Ambrosio, E., Higuera-Matas, A., 2020. The role of the mTOR pathway in models of drug-induced reward and the behavioural constituents of addiction. J Psychopharmacol, 269881120944159. 10.1177/0269881120944159 32854585

[321] Vangindertael J, Camacho R, Sempels W, Mizuno H, Dedecker P, Janssen KPF (2018). An introduction to optical super-resolution microscopy for the adventurous biologist. Methods Appl Fluoresc.

[322] Vegeto E, Belcredito S, Etteri S, Ghisletti S, Brusadelli A, Meda C, Krust A, Dupont S, Ciana P, Chambon P, Maggi A (2003). Estrogen receptor-alpha mediates the brain antiinflammatory activity of estradiol. Proc Natl Acad Sci U S A.

[323] Vegeto E, Belcredito S, Ghisletti S, Meda C, Etteri S, Maggi A (2006). The endogenous estrogen status regulates microglia reactivity in animal models of neuroinflammation. Endocrinology.

[324] Wajant H, Pfizenmaier K, Scheurich P (2003). Tumor necrosis factor signaling. Cell Death Differ.

[325] Wang X, Loram LC, Ramos K, de Jesus AJ, Thomas J, Cheng K, Reddy A, Somogyi AA, Hutchinson MR, Watkins LR, Yin H (2012). Morphine activates neuroinflammation in a manner parallel to endotoxin. Proc Natl Acad Sci U S A.

[326] Ward SJ, Rasmussen BA, Corley G, Henry C, Kim JK, Walker EA, Rawls SM (2011). Beta-lactam antibiotic decreases acquisition of and motivation to respond for cocaine, but not sweet food, in C57Bl/6 mice. Behav Pharmacol.

[327] Warden AS, Azzam M, DaCosta A, Mason S, Blednov YA, Messing RO, Mayfield RD, Harris RA (2019). Toll-like receptor 3 activation increases voluntary alcohol intake in C57BL/6 J male mice. Brain Behav Immun.

[328] Weinhard L, di Bartolomei G, Bolasco G, Machado P, Schieber NL, Neniskyte U, Exiga M, Vadisiute A, Raggioli A, Schertel A, Schwab Y, Gross CT (2018). Microglia remodel synapses by presynaptic trogocytosis and spine head filopodia induction. Nat Commun.

[329] Wetherill RR, Franklin TR, Allen SS (2016). Ovarian hormones, menstrual cycle phase, and smoking: a review with recommendations for future studies. Curr Addict Rep.

[330] Wheeler D, Knapp E, Bandaru VV, Wang Y, Knorr D, Poirier C, Mattson MP, Geiger JD, Haughey NJ (2009). Tumor necrosis factor-alpha-induced neutral sphingomyelinase-2 modulates synaptic plasticity by controlling the membrane insertion of NMDA receptors. J Neurochem.

[331] White J, Kivimaki M, Jokela M, Batty GD (2017). Association of inflammation with specific symptoms of depression in a general population of older people: The English Longitudinal Study of Ageing. Brain Behav Immun.

[332] Wilson NM, Jung H, Ripsch MS, Miller RJ, White FA (2011). CXCR4 signaling mediates morphine-induced tactile hyperalgesia. Brain Behav Immun.

[333] Wu Y, Dissing-Olesen L, MacVicar BA, Stevens B (2015). Microglia: dynamic mediators of synapse development and plasticity. Trends Immunol.

[334] Xiao L, Becker JB (1994). Quantitative microdialysis determination of extracellular striatal dopamine concentration in male and female rats: effects of estrous cycle and gonadectomy. Neurosci Lett.

[335] Xu E, Liu J, Liu H, Wang X, Xiong H (2018). Inflammasome activation by methamphetamine potentiates lipopolysaccharide stimulation of IL-1beta production in microglia. J NeuroImmune Pharmacol.

[336] Yan Y, Nitta A, Koseki T, Yamada K, Nabeshima T (2012). Dissociable role of tumor necrosis factor alpha gene deletion in methamphetamine self-administration and cue-induced relapsing behavior in mice. Psychopharmacology.

[337] Yang XL, Wang X, Shao L, Jiang GT, Min JW, Mei XY, He XH, Liu WH, Huang WX, Peng BW (2019). TRPV1 mediates astrocyte activation and interleukin-1beta release induced by hypoxic ischemia (HI). J Neuroinflammation.

[338] Young K, Morrison H. Quantifying microglia morphology from photomicrographs of immunohistochemistry prepared tissue using ImageJ. J Vis Exp. 2018. 10.3791/57648 PMC6103256 29939190

[339] Yu X, Nagai J, Khakh BS (2020). Improved tools to study astrocytes. Nat Rev Neurosci.

[340] Yu X, Taylor AMW, Nagai J, Golshani P, Evans CJ, Coppola G, Khakh BS (2018). Reducing astrocyte calcium signaling in vivo alters striatal microcircuits and causes repetitive behavior. Neuron.

[341] Zhang Y, Li H, Li Y, Sun X, Zhu M, Hanley G, Lesage G, Yin D (2011). Essential role of toll-like receptor 2 in morphine-induced microglia activation in mice. Neurosci Lett.

[342] Zhang Z, Qin P, Deng Y, Ma Z, Guo H, Guo H, Hou Y, Wang S, Zou W, Sun Y, Ma Y, Hou W (2018). The novel estrogenic receptor GPR30 alleviates ischemic injury by inhibiting TLR4-mediated microglial inflammation. J Neuroinflammation.

[343] Zhao J, Shen S, Dai Y, Chen F, Wang K (2019). Methamphetamine induces intestinal inflammatory injury via nod-like receptor 3 protein (NLRP3) inflammasome overexpression in vitro and in vivo. Med Sci Monit.

[344] Zhao L, Zabel MK, Wang X, Ma W, Shah P, Fariss RN, Qian H, Parkhurst CN, Gan WB, Wong WT (2015). Microglial phagocytosis of living photoreceptors contributes to inherited retinal degeneration. EMBO Mol Med.

[345] Zhong P, Wang W, Yu F, Nazari M, Liu X, Liu QS (2012). Phosphodiesterase 4 inhibition impairs cocaine-induced inhibitory synaptic plasticity and conditioned place preference. Neuropsychopharmacology.

[346] Zhou Y, Danbolt NC (2013). GABA and glutamate transporters in brain. Front Endocrinol (Lausanne).

[347] Zhu R, Bu Q, Fu D, Shao X, Jiang L, Guo W, Chen B, Liu B, Hu Z, Tian J, Zhao Y, Cen X (2018). Toll-like receptor 3 modulates the behavioral effects of cocaine in mice. J Neuroinflammation.

[348] Zlotnik A, Gruenbaum BF, Mohar B, Kuts R, Gruenbaum SE, Ohayon S, Boyko M, Klin Y, Sheiner E, Shaked G, Shapira Y, Teichberg VI. The effects of estrogen and progesterone on blood glutamate levels: evidence from changes of blood glutamate levels during the menstrual cycle in women. Biol Reprod. 2011;84:581–6. 10.1095/biolreprod.110.088120 20980684

